# Unveiling the Mechanisms Ruling the Efficient Hydrogen Evolution Reaction with Mitrofanovite Pt_3_Te_4_

**DOI:** 10.1021/acs.jpclett.1c01261

**Published:** 2021-09-02

**Authors:** Danil W. Boukhvalov, Jia Cheng, Gianluca D’Olimpio, François C. Bocquet, Chia-Nung Kuo, Anan Bari Sarkar, Barun Ghosh, Ivana Vobornik, Jun Fujii, Kuan Hsu, Li-Min Wang, Ori Azulay, Gopi Nath Daptary, Doron Naveh, Chin Shan Lue, Mykhailo Vorokhta, Amit Agarwal, Lixue Zhang, Antonio Politano

**Affiliations:** † College of Science, Institute of Materials Physics and Chemistry, Nanjing Forestry University, Nanjing 210037, P. R. China; ‡ Theoretical Physics and Applied Mathematics Department, Ural Federal University, Mira Street 19, 620002 Ekaterinburg, Russia; § College of Chemistry and Chemical Engineering, Qingdao University, Qingdao 266071, Shandong, P. R. China; ∥ INSTM and Department of Physical and Chemical Sciences, University of L’Aquila, via Vetoio, 67100 L’Aquila (AQ), Italy; ⊥ Peter Grünberg Institut (PGI-3), Forschungszentrum Jülich, 52425 Jülich, Germany; # Jülich Aachen Research Alliance (JARA), Fundamentals of Future Information Technology, 52425 Jülich, Germany; ¶ Department of Physics, National Cheng Kung University, 1 Ta-Hsueh Road, 70101 Tainan, Taiwan; ∇ Department of Physics, Indian Institute of Technology Kanpur, Kanpur, 208016, India; ○ CNR-IOM, TASC Laboratory, Area Science Park-Basovizza, 34139 Trieste, Italy; □ Department of Physics/Graduate Institute of Applied Physics, National Taiwan University, Taipei 10617, Taiwan; ▲ Faculty of Engineering and Institute of Nanotechnology, Bar-Ilan University, Ramat-Gan 52900, Israel; ⬡ Department of Physics and Institure of Nanotechnology, Bar-Ilan University, Ramat-Gan 52900, Israel; & Charles University, V Holesovickǎch 2, Prague 8, 18000 Prague, Czechia; ● CNR-IMM Istituto per la Microelettronica e Microsistemi, VIII strada 5, I-95121 Catania, Italy

## Abstract

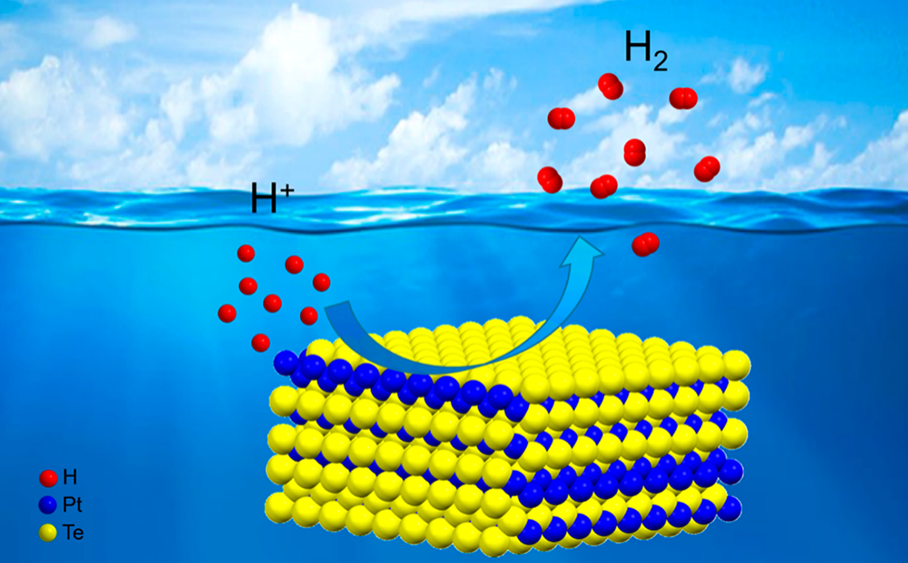

By means of electrocatalytic tests, surface-science techniques and density functional theory, we unveil the physicochemical mechanisms ruling the electrocatalytic activity of recently discovered mitrofanovite (Pt_3_Te_4_) mineral. Mitrofanovite represents a very promising electrocatalyst candidate for energy-related applications, with a reduction of costs by 47% compared to pure Pt and superior robustness to CO poisoning. We show that Pt_3_Te_4_ is a weak topological metal with the  invariant, exhibiting electrical conductivity (∼4 × 10^6^ S/m) comparable with pure Pt. In hydrogen evolution reaction (HER), the electrode based on bulk Pt_3_Te_4_ shows a very small overpotential of 46 mV at 10 mA cm^–2^ and a Tafel slope of 36–49 mV dec^–1^ associated with the Volmer–Heyrovsky mechanism. The outstanding ambient stability of Pt_3_Te_4_ also provides durability of the electrode and long-term stability of its efficient catalytic performances.

In the prospect of sustainable energy, molecular hydrogen represents a promising carbon-free and renewable energy carrier. In particular, HER enables achieving ultrapure hydrogen.^1−5^ Specifically, HER is the cathodic reaction in electrochemical water splitting (2H_2_O → 2H_2_ + O_2_).^1,2,5^ Considering its crucial technological relevance, the identification of efficient electrocatalysts for HER with high activity, cheapness and long-term stability represents one of the most important open challenges in electrochemistry.^1−5^ To date, Pt is generally assumed to be the state-of-the-art HER electrocatalyst, owing to its nearly zero overpotential, relatively low Tafel slope, and good stability.^6^ Regrettably, the abundance of Pt on the crust of Earth is only 0.005 ppm by weight,^7^ and consequently, the corresponding cost is about $ 900/oz, which significantly hinders its extensive technological use. Moreover, Pt electrodes are inevitably affected by CO poisoning,^8^ due to the high reactivity of Pt sites toward CO.^9^ Furthermore, the typical cathode Pt/C catalyst suffers from possible toxicity^10^ and dissolution during the potential cycling with reduction in its active surface area.^10^

Subsequently, the scientific community is engaged in the quest for alternative catalysts. A possible solution relies on reducing Pt loading in Pt-based alloys,^11,12^ without sacrificing the catalytic activity. Among Pt alloys, Pt-based layered materials deserve particular attention^10,13^ for catalysis. As a matter of fact, being van der Waals materials, they can be thinned down to atomic thickness by liquid-phase exfoliation, with the possibility to achieve nanosheets with high surface area and superior catalytic activity.^14−16^ Especially, the transition-metal dichalcogenides (TMDs) PtX_2_ (X = S, Se, Te), which crystallize in the same structure as the naturally occurring mineral “moncheite” hosts pronounced chemical/structural flexibility.^17,18^

Among Pt-based layered materials, PtTe_2_ has been found to be the most performing electrode material for HER, with an overpotential of 0.54 V and a Tafel slope of 110 mV·dec^–1 13^. Notably, PtTe_2_ becomes successfully activated for HER upon oxidative treatment.^19^ Adsorption via the Volmer process is the rate-determining step for the electrochemically treated noble-metal tellurides.^19^ In addition, recent calculations evidence that void-containing Pt_4_Te_7_ could exhibit outstanding HER catalytic performance, due to the Gibbs free energy near to zero (<0.07 eV).^20^ Unfortunately, all Pt-containing chalcogenides investigated to date are semimetals (PtTe_2_^21^ and PtSe_2_^22^) or semiconductors (PtS_2_^23^). However, it is well-known that H adsorption depends on the density of states (DOS) around the Fermi level,^24−26^, so that metallic systems would the most suitable ones for effective HER. On the other hand, surface oxidation leads to the metal–semiconductor (or insulator) transition, which usually decreases catalytic performance. Hence, the combination of features in electronic structure around Fermi level and chemical stability of metallic centers is essential for catalytic application of metallic materials.

Mitrofanovite has been recently discovered as a new natural mineral in the Kola Peninsula, Russia.^27^ Very recently, Bae et al. have reported the synthesis of mitrofanovite Pt_3_Te_4_ nanocrystals on a metallic molybdenum ditelluride (MoTe_2_) template by an electrochemical method, which shows good HER performance.^28^ However, the relationship between the HER activity and the physicochemical properties of Pt_3_Te_4_ remains ambiguous, also considering the improper analysis of electronic band structure, with subsequent misleading theoretical model (see Section S16 in the Supporting Information for more details). Furthermore, such synthesis procedure is unsuitable for large-scale production and fails in reproducibility, due to its complexity. Note that the direct tellurization of Pt layer will produce PtTe_2_ instead of Pt_3_Te_4_ as the enthalpy of formation of PtTe_2_ is the lowest in the Pt–Te system.^29,30^

Single-crystal Pt_3_Te_4_ bulk samples could provide a more suitable platform to explore the catalytic activity of mitrofanovite and highlight its peculiarities. Here, we clarify the physicochemical mechanisms ruling the high efficiency in HER of mitrofanovite Pt_3_Te_4_ bulk crystals, which displays an overpotential as low as 46 mV at a current density of 10 mA cm^–2^ and a Tafel slope of 36–49 mV·dec^–1^ (depending on surface treatments). Moreover, we discover that Pt_3_Te_4_ bulk crystals exhibit high electrical conductivity (3.9 × 10^6^ S/m), which contributes to its high electrocatalytic activity by decreasing the energy cost of delivery of the electrons from source to catalytic sites on the surface. Hence, mitrofanovite represents a suitable candidate as Pt-based layered electrocatalyst, with a reduced loading of Pt, implying a decrease of costs by 47% compared to pure Pt.

Mitrofanite belongs to the trigonal space group *R*3̅*m* (No. 166), and it has a hexagonal structure. Bulk Pt_3_Te_4_ structure is formed by alternating Pt_2_Te_2_ and PtTe_2_ sublayers (constituting a septuple layer) stacked together via weak van der Waals force along the vertical direction (Figure 1a–c). In the PtTe_2_ (Pt_2_Te_2_) sublayer, one (two) Pt atomic layers is (are) sandwiched between two Te atomic layers. The calculated value of binding energy between Pt_3_Te_4_ septuple layers (Pt_2_Te_2_ + PtTe_2_ sublayers) in the bulk is 70.39 kJ per formula unit, while that one between Pt_2_Te_2_ and PtTe_2_ sublayers is 78.13 kJ per formula unit. Calculated values are slightly higher than interlayer van der Waals bond in bulk PtTe_2_ (67.24 kJ per formula unit), but in both cases exfoliation in nanosheets is feasible, with the possibility to produce functional inks by liquid-phase exfoliation.

**Figure 1:**
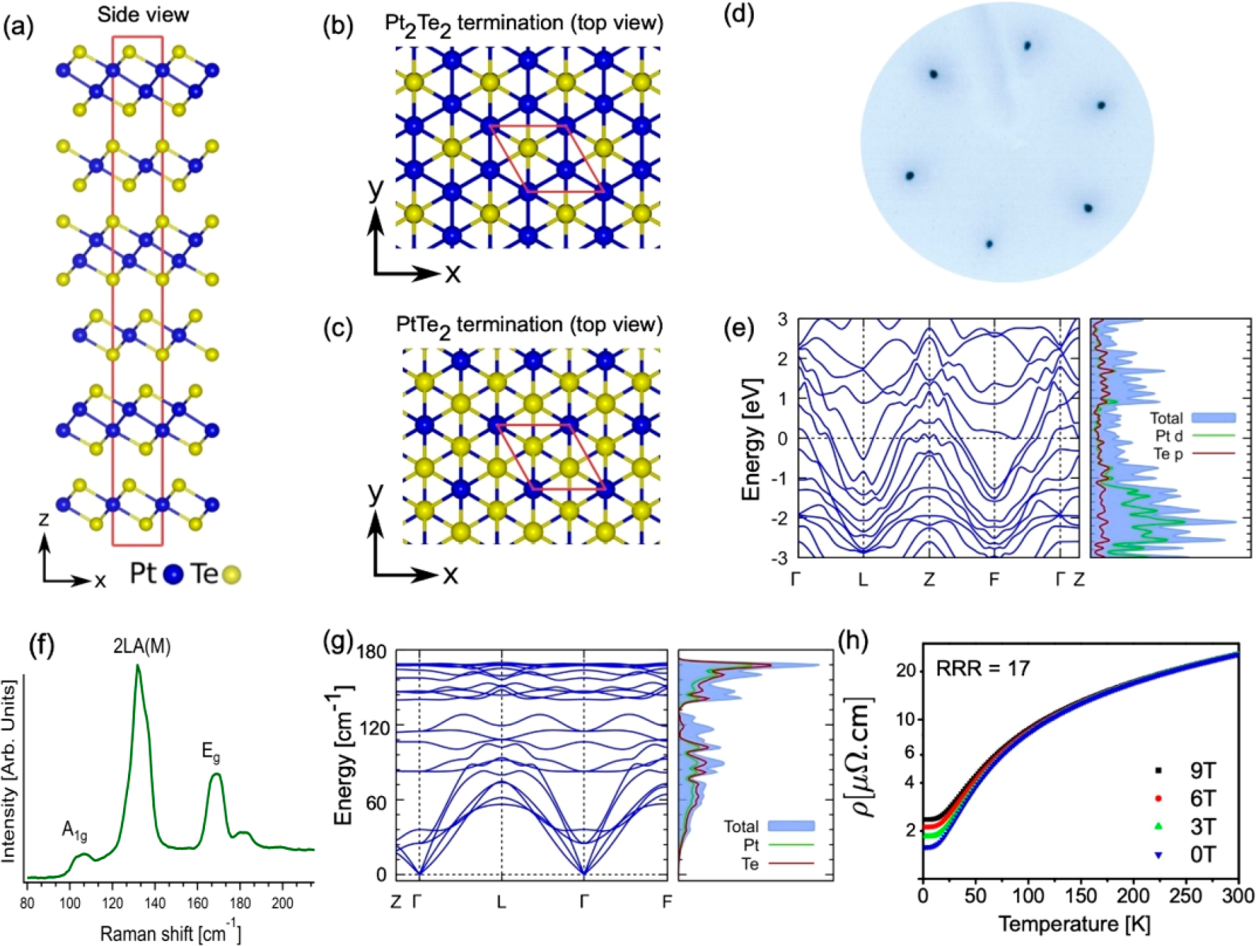
(a) Side view of the layered Pt_3_Te_4_ crystal structure, with alternate PtTe_2_ and Pt_2_Te_2_ layers stacked along the vertical direction. Panels b and c show the top view of the Pt_3_Te_4_ surface with (b) Pt_2_Te_2_ and (c) PtTe_2_ terminations, respectively. (d) LEED pattern obtained at an energy of 100 eV. (e) Band structure and the corresponding density of states. (f) Raman spectrum acquired using a laser with 632.8 nm wavelength. (g) Theoretical phonon dispersion in the primitive unit cell along with the phonon density of states. (h) Resistivity as a function of temperature, for four different values of the magnetic field, ranging from 0 to 9 T.

The bulk Pt_3_Te_4_ single crystal displays a hexagonal surface symmetry, as evinced by the analysis of the low-energy electron diffraction (LEED) pattern (Figure 1d), and three Raman-active modes around 107, 132, and 169 cm^–1^ (Figure 1f). Such bands are assigned to , E_g_^1^ and E_g_^2^ phonons, on the basis of DFT calculations (see Section S4 of the Supporting Information for more details) of phonon dispersion (Figure 1g, left panel) and the corresponding density of states (DOS, Figure 1g, right panel).

Further information on structural properties is reported in the Supporting Information, with synchrotron-based X-ray diffraction (XRD) experiments (Figure S4) and high-resolution transmission electron microscopy (HR-TEM) images (Figure S6). Pt_3_Te_4_ crystallizes in the space group *R*3̅*m* (166), with lattice parameters *a* = 3.99 Å and *c* = 35.40 Å.

The electronic band-structure of Pt_3_Te_4_, including spin–orbit coupling (SOC), is shown in Figure 1e, along with the associated DOS (see Supporting Information, Section S2, for more details on the theoretical model). The orbital-projected DOS clearly shows that the Pt-d and the Te-p orbitals contribute almost equally to the DOS near the Fermi energy, while the contribution from the Pt-d orbitals is predominant for binding energies (BEs) between 1 and 3 eV. The inspection of the band structure reveals that Pt_3_Te_4_ has a metallic nature, hosting several large electron pockets and a small hole pocket at the Fermi energy. Precisely, we find that Pt_3_Te_4_ is a weak topological metal with the  invariant (see Supporting Information, Section S13). The metallic nature is experimentally confirmed by the experimental valence band (Supporting Information, Figure S2) and by transport experiments (Figure 1h). The *ab*-plane resistivity exhibits a clear metallic behavior with the residual resistivity ratio (RRR = ρ(300 K)/ρ(2 K)) ∼ 17. Resistivity at room temperature is ∼26 μΩ·cm. Correspondingly, electrical conductivity is estimated to be ∼4 × 10^6^ S/m, i.e., a value comparable with that of pure Pt (9.4 × 10^6^ S/m^31^). This high value of the electrical conductivity arises from four pairs of bands, which lead to multiple Fermi pockets in the Brillouin zone (see Supporting Information, Figure S13 for more insights on experimental and theoretical band structure). We also evaluated the carrier concentration *n* to be ∼2 × 10^21^ cm^–3^ by transport experiments, with mobility of the electrons (see Hall experiments in Supporting Information, Section S7).

As a recently discovered natural mineral, the electrochemistry of Pt_3_Te_4_ remains unknown. We directly probed its catalytic activity by testing its electrochemical behavior. Specifically, the cyclic voltammetry tests were carried out in both anodic and cathodic directions in pH 7.0 phosphate-buffered saline electrolyte, by directly using Pt_3_Te_4_ bulk plate as the working electrode. In the anodic sweeps (Figure 2a), two small oxidation peaks appear around 0.49 and 0.92 V (vs Ag/AgCl) within the electrochemical stability window of the electrolyte, which should be ascribed to the oxidation of Te element in Pt_3_Te_4_, forming thermodynamically stable Te species with higher valence (namely, TeO_2_). As Pt_3_Te_4_ is composed of Pt_2_Te_2_ and PtTe_2_ sublayers (Figure 1a–c), these two oxidation peaks represent the different chemical states of the Te element in Pt_3_Te_4_ (see also Te-3d core levels in Figure 3b). Congruently, two small reduction peaks appear at around −0.11 and −0.51 V (vs Ag/AgCl), corresponding to the reduction of Te species formed in the initial anodic sweep. The cathodic electrochemical behavior of Pt_3_Te_4_ (Figure 2b) is very similar to the anodic one, except that the reduction peak at around −1.00 V (vs Ag/AgCl) in the first cathodic sweep is much more apparent, due to the electrochemical reduction of the accumulated surface oxide layer in Pt_3_Te_4_ in air. Compared with the electrochemistry of PtTe_2_,^13,19^ two major differences can be distinguished between Pt_3_Te_4_ and PtTe_2_. First, there are two pairs of redox peaks for Te element in Pt_3_Te_4_, meanwhile there is only one pair of redox peaks for Te element in PtTe_2_. Second, although in neutral conditions, for values of the potential smaller than −1.35 V (vs Ag/AgCl), the hydrogen evolution becomes prominent on Pt_3_Te_4_, meanwhile it is not evident on PtTe_2_ up to more negative values of the potential (−1.8 V vs Ag/AgCl^13,19^), indicating a very promising HER catalytic activity of Pt_3_Te_4_. The above results illustrate the intrinsic and unique redox behaviors of Pt_3_Te_4_ for potentials applied in neutral conditions.

**Figure 2:**
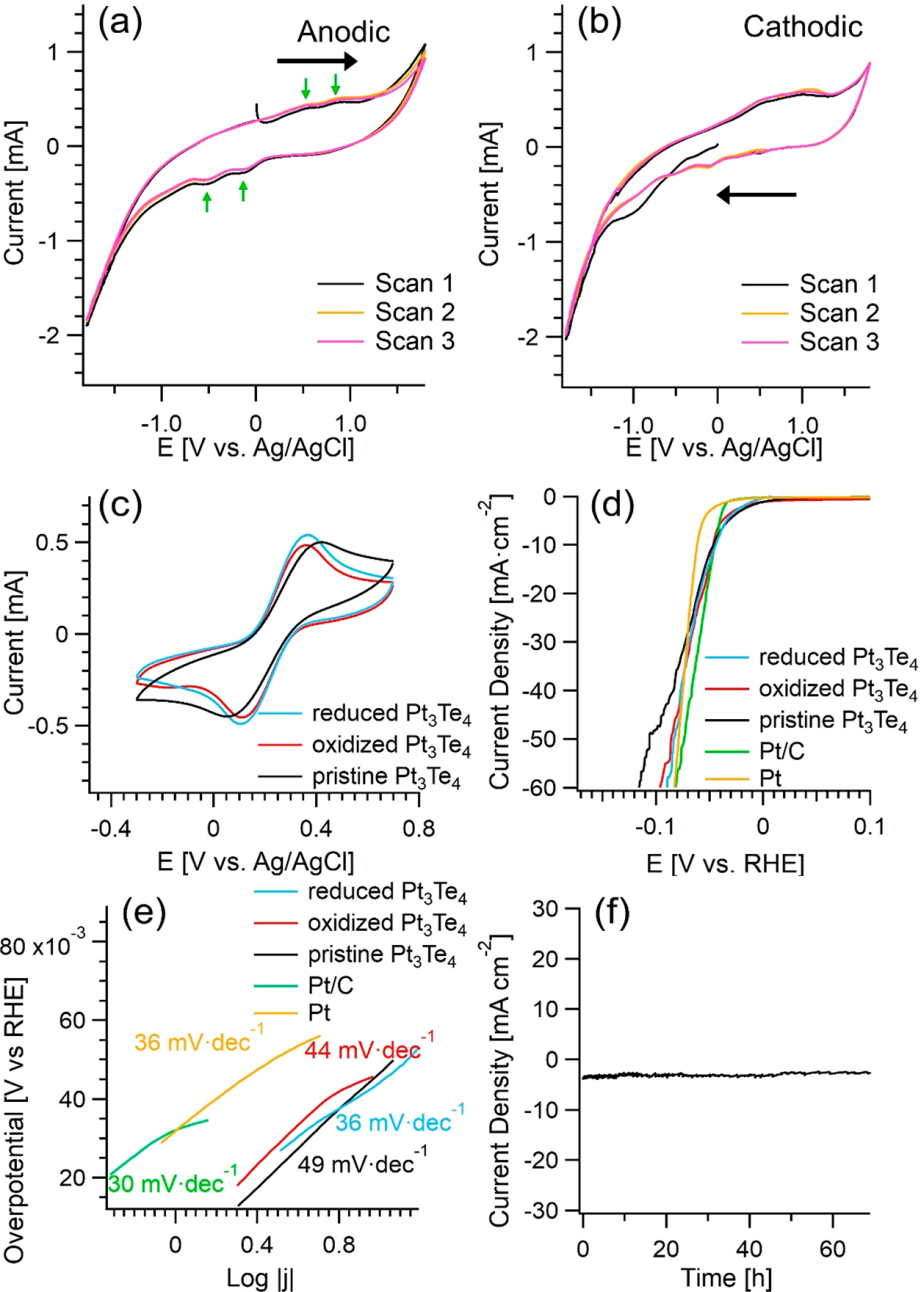
(a) Anodic and (b) cathodic cyclic voltammograms of Pt_3_Te_4_ in 0.05 M phosphate-buffered saline electrolyte (pH 7.0) at a scan rate of 50 mV s^–1^. (c) Cyclic voltammograms of the pristine and the electrochemically treated Pt_3_Te_4_ in 0.1 M KCl solution containing 5 mM [Fe(CN)_6_ ]^3–/4–^ at a scan rate of 50 mV s^–1^. (d) Linear sweep voltammetry curves and (e) the corresponding Tafel plots of Pt_3_Te_4_ and Pt/C catalysts in 0.5 M H_2_SO_4_ solution at a scan rate of 2 mV s^–1^. (f) Chronopotentiometric curve without *iR* correction for bulk Pt_3_Te_4_ in 0.5 M H_2_SO_4_ at a potential of −0.053 V (vs RHE).

**Figure 3:**
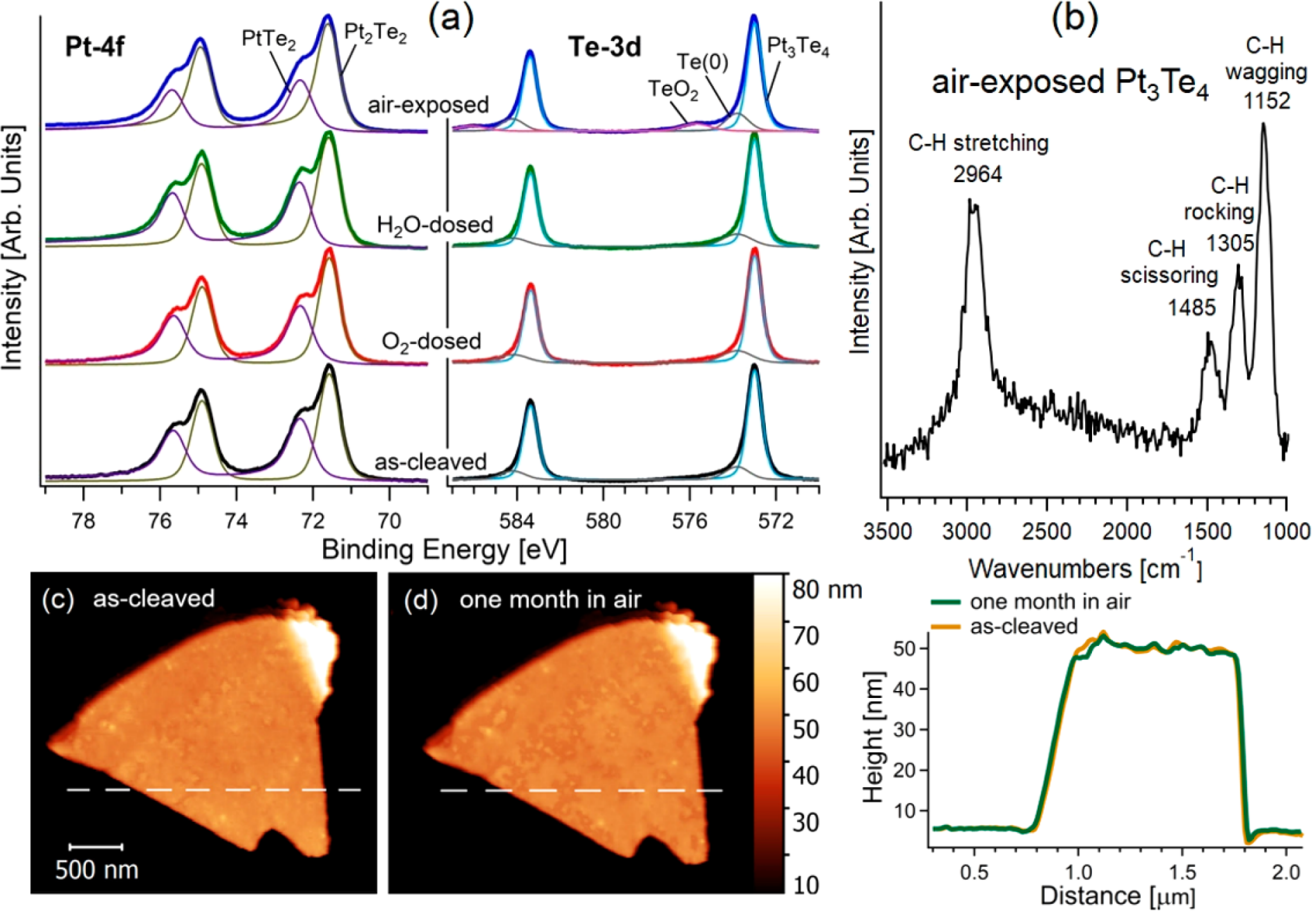
(a) Pt-4f and Te-3d core levels for the as-cleaved Pt_3_Te_4_ surface and its modifications upon exposure to 10^10^ L of O_2_ and H_2_O and, moreover, after storage in air. (b) Vibrational spectrum of air-exposed Pt_3_Te_4_, obtained by high-resolution electron energy loss spectroscopy with an impinging energy of 6 eV. (c, d) Morphological evolution of a Pt_3_Te_4_ flake of 70 nm from (c) sample preparation up to (d) a prolonged exposure to air (one month). (e) Height profile as a function of the distance along dashed white lines in panels c and d. Prolonged storage in air up to 1 month did not provide noticeable morphological changes.

Since a fast rate of heterogeneous electron transfer is one of the prerequisites for a high-performance electrocatalyst, the heterogeneous electron transfer behavior of redox probe on Pt_3_Te_4_ deserves investigation. Considering the observation that Pt_3_Te_4_ undergoes redox behavior under different applied potentials, both the pristine Pt_3_Te_4_ and the same system modified by different electrochemical treatments were investigated with more details. Herein, commonly used [Fe(CN)_6_]^3-/4-^ is selected as the redox probe. Definitely, both the electrochemical treatments, i.e., (i) the oxidizing treatment at a potential of 1.3 V (vs Ag/AgCl) for 5 min and (ii) the reducing treatment at a potential of −1.5 V (vs Ag/AgCl) for 5 min, have a positive influence (Figure 2c). The cyclic voltammogram curves of [Fe(CN)_6_]^3-/4-^ on the pristine, oxidized and reduced Pt_3_Te_4_ are similar. Interestingly, the peak-to-peak separations become even smaller after electrochemical treatments, meaning that the electrochemical redox behavior of [Fe(CN)_6_]^3-/4-^ on the oxidized and reduced Pt_3_Te_4_ are even more reversible.

In addition, the Nyquist plots of the pristine and the electrochemically treated Pt_3_Te_4_ were tested in 0.1 M KCl solution containing 5 mM [Fe(CN)_6_ ]^3–^/^4–^ (Supporting Information, Figure S17). Fitting procedure reveals the charge transfer resistance (*R*_ct_) of the pristine, oxidized and reduced Pt_3_Te_4_ samples to be 102.9, 84.2, and 76.5 Ω, respectively. This result indicates the slightly faster charge transfer kinetics in oxidized and reduced Pt_3_Te_4_ than in pristine Pt_3_Te_4_, consistently with the cyclic voltammetry test.

To correlate the observed modifications in HER activity upon electrocatalytic treatment with changes in physicochemical and electronic properties, mitrofanovite modified by electrochemical treatments was characterized by means of XPS investigations (Supporting Information, Figure S11). Specifically, the oxidation treatment implies the emergence of Te(0) (Te-3d_5/2_ peak at 573.3 eV) and TeO_2_ components (38% of the total spectral area with Te-3d_5/2_ peak at 575.5 eV). Upon reduction treatment, the TeO_2_ component is reduced by 70%. The analysis of Pt-4f reveals that the oxidation treatment also introduces PtO_2_ species (14% of the total spectra area, Pt-4f_7/2_, at BE = 74.0 eV). Corresponding microscopical data are reported in the Supporting Information, Figure S12. It is important to point out that XPS data clarify that no Te dissolution is induced by electrochemical treatments, as evidenced by the quantitative analysis of the Pt/Te ratio.

These observations indicate the unique electrochemical and structural characteristics of Pt_3_Te_4_, which might make it be an effective electrocatalyst under different electrochemical circumstances.

Then, the electrocatalytic performance of bulk Pt_3_Te_4_ toward HER was evaluated by linear sweep voltammetry (LSV) in 0.5 M H_2_SO_4_ solution. For the sake of comparison, the Pt foil electrode was also tested under identical conditions. As shown in Figure 2d, the polarization curve indicates that the HER performance of bulk Pt_3_Te_4_ is very remarkable. When the current density reaches 10 mA cm^–2^, the HER overpotentials for Pt_3_T_4_ is only 46 mV, which is very close to than that of Pt electrode. A similar value was also reported for Pt_3_Te_4_ nanocrystals,^28^ in spite of the reduced surface-to-volume ratio in bulk compared to nanocrystals in ref (28). Moreover, all the Pt_3_T_4_ samples (both pristine one and those ones modified by electrochemical treatments) show almost identical catalytic behavior at the low overpotential region. This finding highlights the unprecedented HER catalytic activity of bulk Pt_3_Te_4_, which is comparable to Pt foil and greatly surpasses all the reported group 10 transition-metal chalcogenides, such as PtTe_2_, PdTe_2_, PtSe_2_, and PtS_2_. It is well-known that the Tafel curve can reflect the reaction kinetics on the catalyst. Correspondingly, the Tafel slope can be used to analyze the reaction mechanism. Figure 2e depicts the Tafel slopes for all the Pt_3_Te_4_ samples and for the Pt foil electrode. For Pt, the Tafel slope in the low overpotential region is estimated to be 36 mV dec^–1^, indicating that the Tafel step of HER is the rate-determining step on Pt. For Pt_3_Te_4_, the calculated Tafel slopes range between 36 and 49 mV dec^–1^. This finding indicates that HER kinetics on Pt_3_Te_4_ is very fast, and it follows a Volmer–Heyrovsky mechanism, in which the Heyrovsky step is the rate-determining step. All the Pt_3_Te_4_-based samples (pristine, oxidized, and reduced) show the similar LSV curves. Remarkably, the Tafel slopes of the oxidized and reduced Pt_3_Te_4_ are even smaller than that of the pristine Pt_3_Te_4_. Accordingly, it is evident that the electrochemical treatments can further improve the HER catalytic activity of Pt_3_Te_4_, which may be related to the enhanced heterogeneous electron transfer capability of Pt_3_Te_4_ by electrochemical treatments.

The superior catalytic activity of Pt_3_Te_4_ could be correlated to its morphology revealed by HR-TEM (Supporting Information, Figure S6). Explictly, the occurrence of different orientation of crystallographic axis in grains implies the formation of multiple grain boundaries with atomic structure closer to defects in Pt_3_Te_4_ surfaces. Such grain boundaries represent additional active sites (beyond Te vacancies), which are expected to provide a noticeable enhancement of catalytic performance.

The electrochemical stability is one of the crucial aspects in assessing the electrocatalytic performance of electrocatalysts. Specifically, the chronopotentiometric curve of Pt_3_Te_4_ at a potential of −0.053 V (vs RHE) in H_2_SO_4_ shows negligible attenuation in a time scale extended up to 69 h (Figure 2f), validating the outstanding chemical and electrocatalytic stability of Pt_3_Te_4_. Conversely, it has been reported that PtTe_2_ suffers from very low HER kinetics and, moreover, its catalytic performance changes by the electrochemical treatments.^13,19^

To get more detailed information on the stability of Pt_3_Te_4_, we carried out a surface-science investigation regarding its chemical reactivity in oxidative and humid environments, including ambient atmosphere. The as-cleaved surface shows a splitting of the Pt-4f core level with two different doublets with *J* = ^7^/_2_ components at BEs of 71.5 and 72.3 eV, respectively. On the other hand, Te-3d has two doublets with the *J* = ^5^/_2_ component at 573.0 and 573.8 eV. To understand the splitting of both Pt-4f and Te-3d core levels, one should consider that the Pt_2_Te_2_ subunit has two different chemical environments for Pt atoms corresponding to Pt–Pt and Pt–Te bonds. Similarly, Te atoms also have two environments, corresponding to Pt–Te and Te–Te bonds. To assign the two spectral components, we computed core-level shifts based on charge distribution in the Pt_2_Te_2_ subunit, finding that the charge on Pt sites in Pt–Te bonds is reduced by 0.363 electrons compared to Pt–Pt bonds, corresponding to a core-level shift by 0.8 eV.

No change in Pt-4f and Te-3d are observed after dosing 10^10^ L of O_2_ and H_2_O on as-cleaved Pt_3_Te_4_ (with 1 L = 10^–6^ Torr·s). The exposure to air only affects the Te-3d core level, with the emergence of new features with *J* = ^5^/_2_ component at BE of 575.7 and 573.8 eV, corresponding to TeO_2_ and Te(0), respectively. Therefore, the outermost Te surface layer evolves into a TeO_2_ skin, whose thickness was estimated to be (0.7 ± 0.2) nm, according to quantitative XPS estimations^32−34^ (see Supporting Information, Section S11 for details). The inspection of survey XPS spectra (Supporting Information, Figure S1) also indicates the presence of carbonaceous species on the air-exposed surface. To get more insights on the nature of these C-containing adsorbates, we carried out a vibrational investigation on the air-exposed surface, which revealed the presence of CH*x* species. As a matter of fact, the vibrational spectrum exhibited the various infrared-active modes of CH*x* species^35−37^ from airborne contamination:^38^ (i) wagging at 1152 cm^–1^; (ii) rocking at 1305 cm^–1^; (iii) scissoring at 1485 cm^–1^; (iv) stretching at 2964 cm^–1^, respectively.

The ambient stability was also assessed by a morphological investigation by atomic force microscopy (AFM) in a time scale extended up to one month. The AFM experiments demonstrate that exposure to air did not modify the morphology of the Pt_3_Te_4_ surface (Figure 3, parts c and d), as established by the minimal changes in the height profile along a specific direction (Figure 3e). Similar conclusions can be inferred from the SEM analyses of the post-mortem Pt_3_Te_4_-based electrode, reported in Figure S8 of the Supporting Information, which does not show any noticeable change in the morphology compared to the as-prepared electrode (Supporting Information, Figure S7).

For the sake of completeness, in Section S4 of the Supporting Information, we also report a post-mortem XPS analysis on the Pt_3_Te_4_ electrode (Supporting Information, Figure S3), which further supports the long-term stability of the electrode in the adopted solvents even after HER tests. Explicitly, the post-mortem XPS analysis only revealed residuals traces of Fe(CN)_6_ and H_2_SO_4_ solvents.

Experimental results were validated by density functional theory (DFT). First, we checked the formation energies of Te vacancies on the two different terminations of Pt_3_Te_4_. For the modeling of the vacancies, we removed one Te atom from the center of top layer of 3 × 3 supercell. We found values as high as 1.55 and 2.35 eV for the PtTe_2_-terminated and Pt2Te_2_-terminated Pt_3_Te_4_ surfaces (Figure 4a). The value of the formation energy for PtTe_2_-terminated surface is almost the same as for bulk PtTe_2_ (1.68 eV). Therefore, the probability of formation of the Te vacancies is moderate.

**Figure 4:**
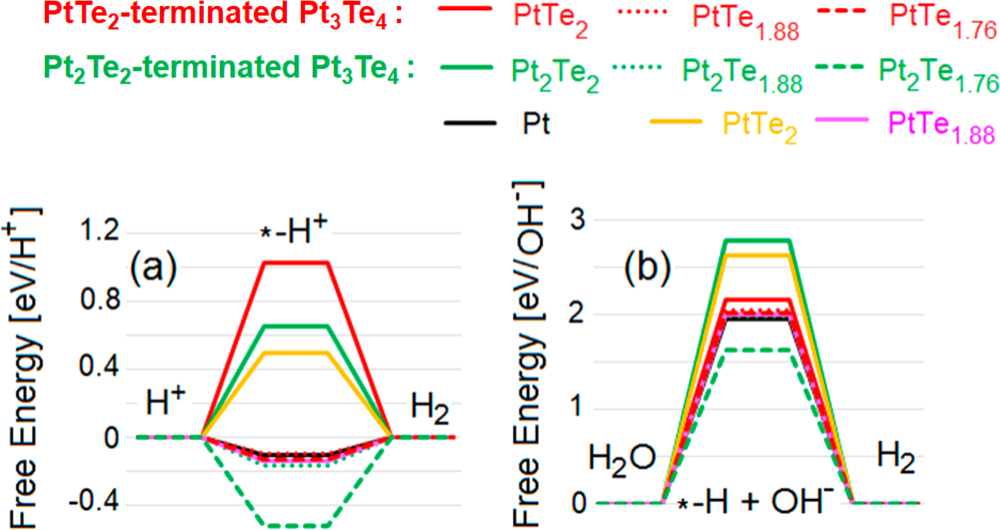
Free energy diagrams for HER in (a) acidic and (b) alkali media on various surfaces of Pt_3_Te_4_. Red lines denote results for the PtTe_2_-terminated Pt_3_Te_4_ surface, while green lines are related to the Pt_2_Te_2_ surface termination of Pt_3_Te_4_. Results for both defect-free and defective surfaces are shown. For the sake of comparison, also values for bulk Pt, PtTe_2_, and PtTe_1.88_ are reported. The symbol “*” corresponds to the substrate.

Successively, we assessed the chemical stability of both surface terminations. Calculations reported in Table 1. I demonstrate that, in the case of CO, only metastable adsorption on defect-free Pt_2_Te_2_ surface is energetically feasible (Δ*G*_ads_ = −1.72 kJ/mol). This makes Pt_3_Te_4_ an ineffective catalyst for CO conversion. It is important to note that Pt_3_Te_4_ behaves differently compared to Pt and other Pt-based alloys, which instead strongly suffer CO poisoning. A direct experimental validation of this theoretical prediction is represented by the comparative investigation reported in Figure S9 of the Supporting Information by means of the experimental technique with the highest sensitivity to CO adsorption,^39^ i.e., HREELS.

**Table 1:** Differential Enthalpy Δ*H*_ads_ and Differential Gibbs Free Energy Δ*G*_ads_ for Physical Adsorption and Differential Enthalpy of Decomposition Δ*H*_dec_ (all in kJ/mol) for Molecular Oxygen and Water on Defect-Free (i) PtTe_2_- and (ii) Pt_2_Te_2_-Terminated Pt_3_Te_4_ Surfaces and, Moreover, in the Nearness of Te Vacancies in These Surfaces (See Figure 1b,c)^a^

adsorbent	surface termination of Pt_3_Te_4_	site	Δ*H*_ads_ [kJ/mol]	Δ*G*_ads_ [kJ/mol]	Δ*H*_dec_ [kJ/mol]
CO	PtTe_2_	defect-free	–15.61	+3.74	–
		Te vacancy	–12.72	+6.63	
	Pt_2_Te_2_	defect-free	–21.07	–1.72	–
		Te vacancy	–12.59	+6.76	
H_2_O	PtTe_2_	defect-free	–25.59	+5.71	+405.20
		Te vacancy	–20.11	+11.19	+428.38
	Pt_2_Te_2_	defect-free	–26.07	+5.23	+173.55
		Te vacancy	–16.48	+14.82	–30.18
O_2_	PtTe_2_	defect-free	–42.62	–31.13	–51.78 (+1.31)
		Te vacancy	–33.29	–21.99	–69.37 (−30.99)
	Pt_2_Te_2_	defect-free	–40.81	–29.51	–98.08 (−27.06)
		Te vacancy	–34.99	–23.69	–162.98 (−73.58)

a In the case of oxygen decomposition, we also report the differential enthalpy of the oxidation of whole surface (in parentheses).

Similarly, our theoretical model predicts that water physisorption is energetically unfavorable for all possible termination, based on the positive values of the differential Gibbs free energy Δ*G*_ads_ for both pristine and defective surfaces. On the other hand, oxygen physisorption at room temperature is energetically feasible, with further decomposition also energetically favorable. Note that the energy gain from oxygen decomposition on the PtTe_2_-terminated Pt_3_Te_4_ surface is about twice smaller than oxygen activation energy (∼130 kJ/mol).^12^ Further oxidation of whole surfaces is energetically unfavorable for the defect-free PtTe_2_-terminated Pt_3_Te_4_ surface, while it is energetically favorable for other surface terminations.

Additionally, we estimated Δ*G*_ads_ for oxygen adsorption at room temperature in the single septuple layer of Pt_3_Te_4_, finding that it is energetically favorable on both the Pt_2_Te_2_ (−42.39 kJ/mol) and PtTe_2_ (−11.99 kJ/mol) sides. The complete oxidation of the single septuple layer is energetically favorable at room temperature on both Pt_2_Te_2_ and PtTe_2_ sides (−52.70 and −7.90 kJ/mol, respectively).

For the sake of completeness, Δ*G*_ads_ for oxygen adsorption at room temperature on the surface of bulk PtTe_2_ is −26.11 kJ/mol, with further complete oxidation energetically favorable (−39.93 kJ/mol)

Thus, oxidation of Pt_*x*_Te_*y*_ naturally occurs in oxidative environments, including air. As the magnitudes of the energies of each steps of oxidation are relatively high and all sites are available (as adsorption of water and carbon monoxide is unfavorable), the oxidation rate of Pt_3_Te_4_ is limited only by oxygen supply. In oxygen-rich environments, oxidation of the Pt_3_Te_4_ surface (for both bulk and atomically thin layers) will take just a few minutes with the formation of a TeO_2_ skin passivating the surface. The reduction of the amount of Te defects increases the robustness to oxidation. Notably, the rather low energy gain from the oxidation enables thermal reduction of the oxidized Pt_3_Te_4_ surface by heating at moderate temperatures (100–200 °C).

To assess the suitability of Pt_3_Te_4_ for catalysis, we modeled HER over both PtTe_2_- and Pt_2_Te_2_-terminated pristine and defective surfaces of bulk Pt_3_Te_4_. As intermediate step of HER in both media corresponds to hydrogen adsorption on catalytic substrate, we simulated adsorption of H on Pt sites. For the sake of comparison, we also performed similar calculations for the surface of the PtTe_2_ crystal. The calculated free energy of HER (Figure 4a,b) demonstrates that both defect-free surfaces of Pt_3_Te_4_ and PtTe_2_ are unsuitable for HER in both acidic and alkali media, without noticeable differences among bulk crystals and nanosheets of Pt_3_Te_4_.

However, the presence of Te vacancies drastically changes the picture. Actually, the free energy of the intermediate step of HER in the nearness of (i) single or double Te-vacancy sites in PtTe_2_-terminated surface (PtTe_1.88_ and PtTe_1.76_) and (ii) single Te-vacancy in Pt_2_Te_2_-terminated surface (Pt_2_Te_1.88_), assumes almost the same values of that corresponding to the Pt(111) surface, usually taken as a standard reference. Note that, in alkali conditions, the energy cost of water decomposition with hydrogen adsorption on Pt_2_Te_1.76_ surface is smaller than that for Pt(111). Thus, the presence of Te vacancies in the surface region plays a pivotal role in the outstanding catalytic performance of Pt_3_Te_4_. Note that the proposed theoretical model of defects-driven catalytic performance of Pt_3_Te_4_ is also valid to describe the contribution of defect-rich areas, such as grain boundaries imaged in HR-TEM images in Figure S6 of the Supporting Information. To unveil the nature of the enhancement of catalytic activity around Te vacancies, we examined the changes in electronic structure of surface Pt atoms before and after hydrogenation. Calculations demonstrate that hydrogenation of Pt atoms does not provides visible changes of its electronic structure (Supporting Information, Figure S14a,b). The formation of Te-vacancy sites leads to the appearance of electronic states in the nearest Pt atoms around the Fermi level. The hydrogenation of such Pt centers eliminates these states with almost complete restoration of the electronic structure of the defect-free sample (Supporting Information, Figure S14c,d). Note that for Pt_2_Te_1.88_ the magnitude of these near-zero states is larger than for PtTe_1.88_, which corresponds to higher catalytic performance of defective Pt_2_Te_2_ surface.

In conclusion, here we have shown that the mitrofanovite (Pt_3_Te_4_) mineral represents a very promising candidate as an electrocatalyst for HER. Mitrofanovite is a weak topological metal with the  invariant, with an electrical conductivity as high as ∼4 × 10^6^ S/m, due to four pairs of bands leading to multiple Fermi pockets in the Brillouin zone. In HER, bulk Pt_3_Te_4_ displays a with a small overpotential of 46 mV at 10 mA cm^–2^, and a Tafel slope of 36–49 mV dec^–1^ associated with the Volmer–Heyrovsky mechanism. Contrary to Pt, which highly suffers CO poisoning, Pt_3_Te_4_ is totally inert toward CO and, moreover, it is stable in both alkaline and acidic environments.

The excellent ambient stability of Pt_3_Te_4_ affords durability of the electrode, with subsequent long-term stability of its efficient catalytic performances.

Compared to pure Pt, which represents the state-of-the-art electrode for HER, mitrofanovite Pt_3_Te_4_ bulk catalyst also provides comparable catalytic performances, with costs of raw materials reduced by 47%. Moreover, the use of mitrofanovite could be extended to other catalytic and energy-related applications.

## Methods

*Single-Crystal Growth.* Single crystals of Pt_3_Te_4_ were grown from the self-flux method. Unlike the case of PtTe_2_,^40^ the growth window of Pt_3_Te_4_ is narrow. The mixtures of high-purity Pt foil and Te ingots with the molar ratio of 51:49 were inserted in an alumina crucible and sealed into an evacuated quartz ampule. The quartz ampule was heated to 1080 °C for 24 h, then slowly cooled to 975 °C at a rate of 1 °C/h. The excess flux was separated by centrifugation above 970 °C and mechanical polishing. Shiny plate-like Pt_3_Te_4_ single crystals were harvested with a dimension of 4 × 3 × 0.4 mm^3^. The flat surface of the crystal corresponds to the (001) plane, as identified by XRD analyses reported in Figure S4 of the Supporting Information.

*Transport.* The transport experiments on the Pt_3_Te_4_ single crystals were carried out using a four-point probe method in a quantum design physical property measurement system (PPMS-9).

*Raman Spectroscopy.* Micro-Raman spectra were obtained at room temperature by means of a LABRAM spectrometer in backscattering configuration, with a 1800 lines/mm diffraction grating and laser spot cross diameter of about 2 μm. The laser source is He–Ne (λ = 632.8 nm). The optical microscope has a 100× MPLAN with numerical aperture of 0.9.

*AFM.* AFM images were obtained using a Digital D5000, Veeco system working in Tapping-mode. The tip has a resonance frequency of 75 kHz.

*Near-Ambient Pressure XPS.* Both UHV and NAP-XPS measurements were performed at Surface Physics Laboratory in Prague using a custom-built (SPECS Surface Nano Analysis GmbH) spectrometer equipped with a high-pressure (NAP) cell, monochromatized Al Kα X-ray source, and hemispherical electron analyzer Phoibos 3500 (see ref (41) for a description of the NAP-XPS equipment). While doing the high-pressure XPS measurements, the as-cleaved sample was inserted into the NAP cell and docked to the analyzer. Then the cell was filled with 1 mbar of O_2_ for 12 h and the XPS spectra were collected in the presence of O_2_ after 2 and 12 h of exposure. The same workflow was used for the H_2_O exposure.

*Theoretical Methods.* Modeling of the atomic structure and energetics of gas adsorption on Pt_3_Te_4_ was carried out using the QUANTUM-ESPRESSO code^42^ and the GGA–PBE functional with van der Waals (vdW) corrections, feasible for the studying of the adsorption of molecules on surfaces.^43,44^ Energy cutoffs of 25 and 400 Ry for the plane-wave expansion of the wave functions and the charge density, respectively, and the 4 × 4 × 3 Monkhorst–Pack *k*-point grid for the Brillouin sampling were used.^45^

For the modeling of the surface, we used slab of two Pt_3_Te_4_ layers each of these layers contain PtTe_2_ and Pt_2_Te_2_ layers (see Figure 1). Note that in the slab of any number of Pt_3_Te_4_ layers on one surface will be Pt_2_Te_2_ layer and on opposite side PtTe_2_ layer. Moreover, we also considered the presence of the Te vacancies in top layer. To imitate contribution from rigid subsurface area of bulk crystals in the modeling of the surfaces, we performed optimization of the only atomic positions. In order to take into account the contribution from flexibility of nanosheets, we performed optimization of both atomic positions and lattice parameters.

The enthalpies of physical adsorption were calculated by the standard formula:
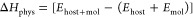
Here, *E*_host_ is the total energy of the surface before adsorption, and *E*_mol_ is the energy of the single molecules of considered species in empty box. In the case of water adsorption, we only considered adsorption from the gaseous phase. Energy of chemical adsorption is defined as difference between the total energy of the system after and before decomposition of physically adsorbed molecule. For the case of physical adsorption, we also evaluated differential Gibbs free energy by the formula
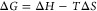
where *T* is the temperature and Δ*S* is the change of entropy after formation molecule–substrate noncovalent bond, which was estimated similar to the gas → liquid transition and hence can be evaluated by the standard formula
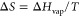
where Δ*H*_vap_ is the empirical enthalpy of vaporization.

Methods to calculate phonons and band structure are reported in Section S3 of the Supporting Information.

*Electrochemical Tests.* Electrochemical tests were carried out on a Bio-Logic VSP-300 electrochemical workstation with a typical three-electrode system, in which a bulk Pt_3_Te_4_ plate, a Pt wire and a saturated Ag/AgCl were used as the working electrode, the counter electrode, and the reference electrode, respectively. The Pt/C electrode was prepared by casting 2 μL of the dispersion ink of 1 mg mL^–1^ Pt/C (20 wt % Pt, purchased from Sigma-Aldrich Chemical Reagent Co., Ltd.) onto a glassy carbon electrode, which was then left to dry in air. The inherent electrochemical behaviors of Pt_3_Te_4_ was in 0.05 M phosphate buffered saline electrolyte (pH 7.0) at a scan rate of 50 mV s^–1^. Electrochemical treatments of Pt_3_Te_4_ were realized by applying a potential of 1.3 V (vs Ag/AgCl) or −1.5 V (vs Ag/AgCl) on Pt_3_Te_4_ electrode for 5 min in 0.05 M phosphate buffered saline electrolyte. The redox behavior of [Fe(CN)_6_]^3-/4-^ on Pt_3_Te_4_ electrodes were measured in 0.1 M KCl solution containing 5 mM [Fe(CN)_6_ ]^3–/4–^ at a scan rate of 50 mV s^–1^. The Nyquist plots of the pristine and the electrochemically treated Pt_3_Te_4_ were tested in 0.1 M KCl solution containing 5 mM [Fe(CN)_6_ ]^3–/4–^ at an open-circuit potential with an amplitude of 5 mV, and the frequency range is 10^6^ to 0.01 Hz. For HER tests, the polarization curves were obtained using LSV in 0.5 M H_2_SO_4_ at a scan rate of 2 mV s^–1^. The chronopotentiometric test was performed in 0.5 M H_2_SO_4_ at a potential of −0.053 V (vs RHE). The potentials in the HER part were calibrated to RHE, according to the following equation:
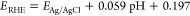


## Supporting Information Available

XPS, SEM, and TEM characterization of samples before and after electrochemical treatment, valence bands XPS spectra, technical details of DFT modeling, calculated infrared and Raman frequencies, electronic and band structures and detailed comparison with previous theoretical calculations, synchrotron X-ray powder diffractions patterns, transport measurements, TEM and SEM images, assessment of CO adsorption by the HREELS technique, determination of the thickness of oxide skin, and Nyquist plots (PDF)
