# Supplementary material for: Unveiling the Mechanisms Ruling the Efficient Hydrogen Evolution Reaction with Mitrofanovite Pt3Te4
Source: J Phys Chem Lett. 2021 Sep 2;12(35):8627–36. doi: 10.1021/acs.jpclett.1c01261 (PMC8436201; doi:10.1021/acs.jpclett.1c01261)
Supplement: Supplementary file 1 — jz1c01261_si_001.pdf [file jz1c01261_si_001.pdf]

Supporting Information for the paper

Unveiling the Mechanisms Ruling the Efficient Hydrogen Evolution Reaction with Mitrofanovite Pt_3Te_4

Danil W. Boukhvalov[1,2], Jia Cheng[3], Gianluca D'Olimpio[4], François C. Bocquet[5,6], Chia-Nung Kuo[7], Anan Bari Sarkar[8], Barun Ghosh[8], Ivana Vobornik[9], Jun Fujii[9], Kuan Hsu[10], Li-Min Wang[10], Ori Azulay,[11] Gopi Nath Daptary[12], Doron Naveh[11], Chin Shan Lue[7], Mykhailo Vorokhta [13], Amit Agarwal[8], Lixue Zhang[3], Antonio Politano[4,14]

[1] College of Science, Institute of Materials Physics and Chemistry, Nanjing Forestry University, Nanjing 210037, P. R. China
[2] Theoretical Physics and Applied Mathematics Department, Ural Federal University, Mira Street 19, 620002 Ekaterinburg, Russia
[3] College of Chemistry and Chemical Engineering, Qingdao University, Qingdao 266071, Shandong, China
[4] INSTM and Department of Physical and Chemical Sciences, University of L'Aquila, via Vetoio, 67100 L'Aquila (AQ), Italy
[5] Peter Grünberg Institut (PGI-3), Forschungszentrum Jülich, 52425 Jülich, Germany
[6] Jülich Aachen Research Alliance (JARA), Fundamentals of Future Information Technology, 52425 Jülich, Germany
[7] Department of Physics, National Cheng Kung University, 1 Ta-Hsueh Road, 70101 Tainan, Taiwan
[8] Department of Physics, Indian Institute of Technology Kanpur, Kanpur, 208016, India
[9] CNR-IOM, TASC Laboratory, Area Science Park-Basovizza, 34139 Trieste, Italy
[10] Department of Physics/Graduate Institute of Applied Physics, National Taiwan University, Taipei 10617, Taiwan
[11] Faculty of Engineering and Institute of Nanotechnology, Bar-Ilan University, Ramat-Gan Israel, 52900
[12] Department of Physics and Institure of Nanotechnology, Bar-Ilan University, Ramat-Gan Israel, 52900
[13] Charles University, V Holesovickăch 2, Prague 8, 18000 Prague, Czechia
[14] CNR-IMM Istituto per la Microelettronica e Microsistemi, VIII strada 5, I-95121 Catania, Italy

S1. X-ray photoelectron spectroscopy

Survey XPS spectra taken for as-cleaved Pt_3Te_4 demonstrate the absence of contaminants, including C and O, in the pristine sample (Fig. S1a). On the other hand, air exposure induces the emergence of additional features related to oxygen and carbon (Fig. S1a), as indicated by O-1s at 530 eV and C-1s around 284 eV (Fig. S1b). While O-1s is evidently related to TeO_2 phases formed upon oxidation in air (see main text, Fig. 3a), attributing the origin of C-1s requires dedicated vibrational experiments, reported in Fig. 3b of the main text. Explicitly, C-1s is related to airborne CHx contamination.

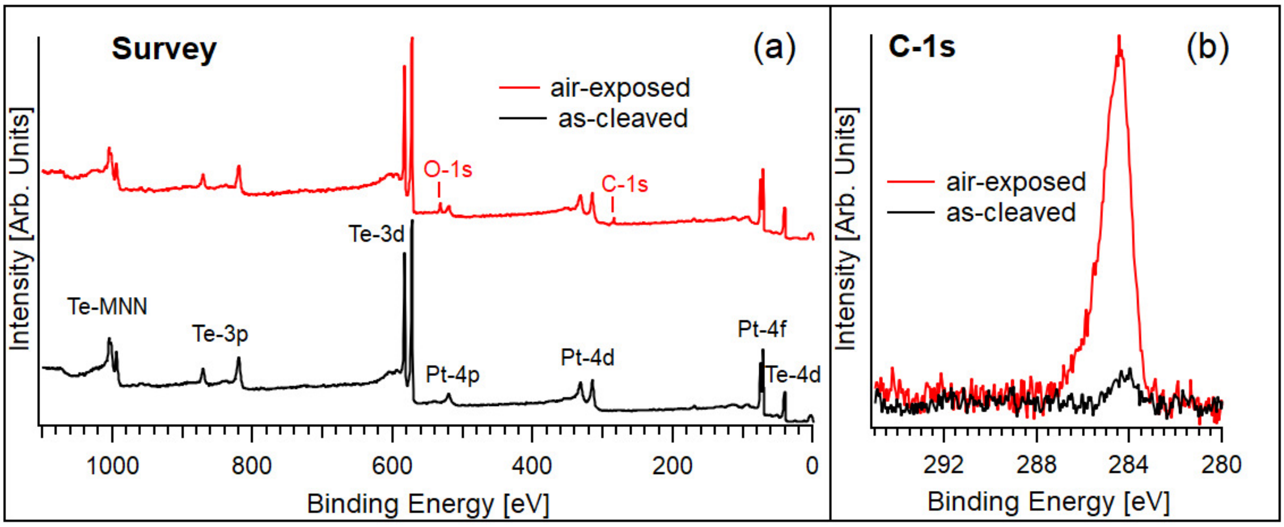

Figure S1. Survey XPS spectra of as-cleaved (black curve) and air-exposed (red-curve) Pt_3Te_4. The C-1s core level in both cases is shown in panel (b).

S2. Valence band

The valence band was measured for pristine Pt_3Te_4 and for the same surface exposed to most common ambient gases and air. Valence-band states survive with negligible modifications even in air-exposed surface.

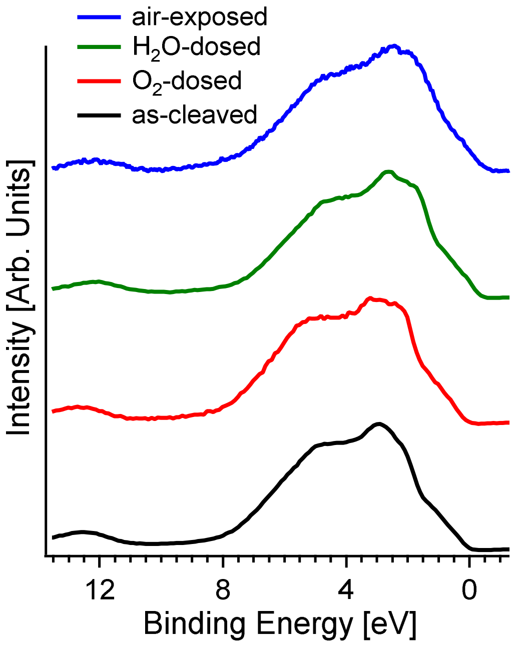

Figure S2. Valence band of pristine Pt_3Te_4 and the same surface after exposure to O_2, H_2O and air. The photon energy is 900 eV. Experiments were carried out at Elettra synchrotron, Trieste.

S3 Details of the DFT calculations:

The electronic band structure and the phonon spectrum were calculated using the Vienna Ab-initio Simulation Package (VASP). [1-3] We used projector augmented wave (PAW) pseudopotentials with a plane wave basis. The kinetic energy cut-off of the plane wave basis was set to 400 eV. In order to perform the Brillouin zone integration, a Γ-centered 12x12x8 *k*-grid is used. The exchange-correlation part of the potential was treated using the generalized gradient approximation (GGA) scheme. [4] We relaxed the atomic positions of the experimental structure until the force on each atom became vanishingly small. Phonon calculations were performed within the "frozen-phonon" method with the help of PHONOPY software. [5]

S4. Theoretical insights on infrared- and Raman-active phonons

The phonon frequencies (in cm^{-1}) at the Γ-point of bulk Pt_3Te_4 are presented in Table S1. The irreducible representations and the optical activity of each of these phonon modes are also tabulated. In the primitive cell of Pt_3Te_4, there are a total of seven atoms, leading to 21 distinct phonon bands. For symmetry, 9 (including three pairs of degenerate modes) of such 21 phonon bands contribute to the Raman activity.

Table S1. Infrared- and Raman-active phonons of Pt_3Te_4. The frequency and the symmetry of each mode are also reported.

Branch number	Frequency (cm^{-1})	Symmetry	Infrared (I) / Raman (R) activity
1-2	0	E_u	-
3	0	A_{2u}	-
4-5	25	E_u	I
6	36	A_{2u}	I
7-8	83	E_g	R
9	106	A_{1g}	R
10-11	115	E_g	R
12	140	A_{2u}	I
13-14	146	E_u	I
15	147	A_{1g}	R
16	157	A_{1g}	R
17	164	A_{2u}	I
18-19	168	E_g	R
20-21	169	E_u	I

S5. Core levels in post-mortem electrode

In the post-mortem Pt_3Te_4-based electrode traces of residual solvents are evident, as indicated by the appearance of N-1s and S-2p core levels, absent in the as-prepared electrode.

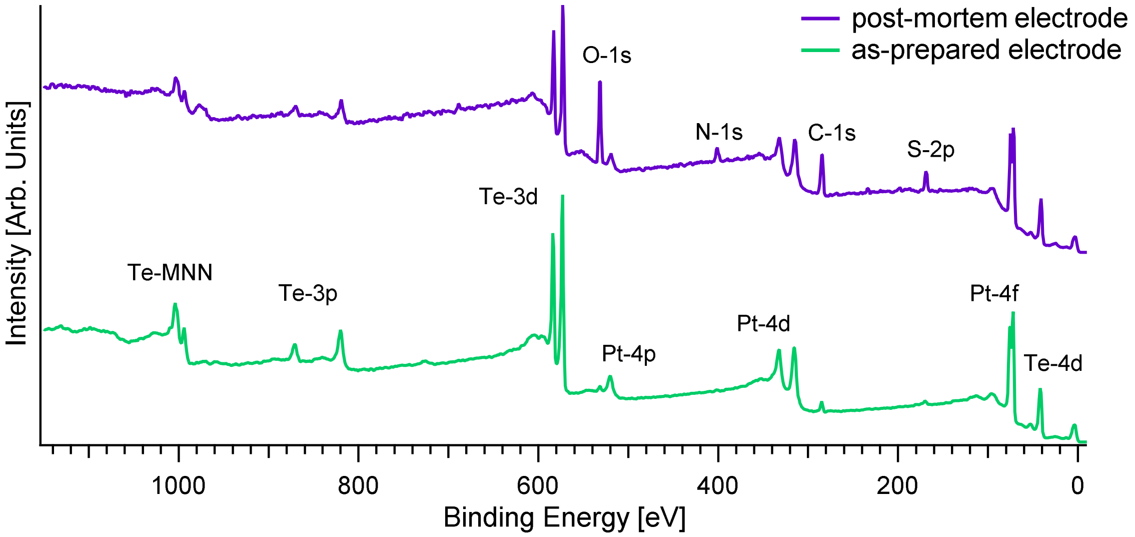

Figure S3. Survey XPS spectra for as-prepared and post-mortem electrodes.

S6. Synchrotron X-ray powder diffraction

The in-house XRD system is insufficient for phase identification and structure analysis due to the malleability of Pt_3Te_4 crystals. To reduce the broadening of peaks and preferential orientation, a synchrotron x-ray powder diffraction (SXRD) experiment was conducted. The SXRD patterns were collected from 100 to 480 K with the MYTHEN detector with 15 keV beam at beam line 09A, Taiwan Photon Source, National Synchrotron Radiation Research Center (NSRRC) in Hsinchu, Taiwan. The single crystal was pulverized and packed in a 0.1 mm borosilicate capillary to minimize the absorption effect. The capillary was kept spinning during data collection for powder averaging.

As shown in Fig. S4a, all diffraction peaks match well with the Pt_3Te_4 structure (ICSD # 41372). Fig. S4b shows the SXRD patterns in the temperature range 100 - 480 K. The absence of extra reflections also confirmed the structural stability of Pt_3Te_4.

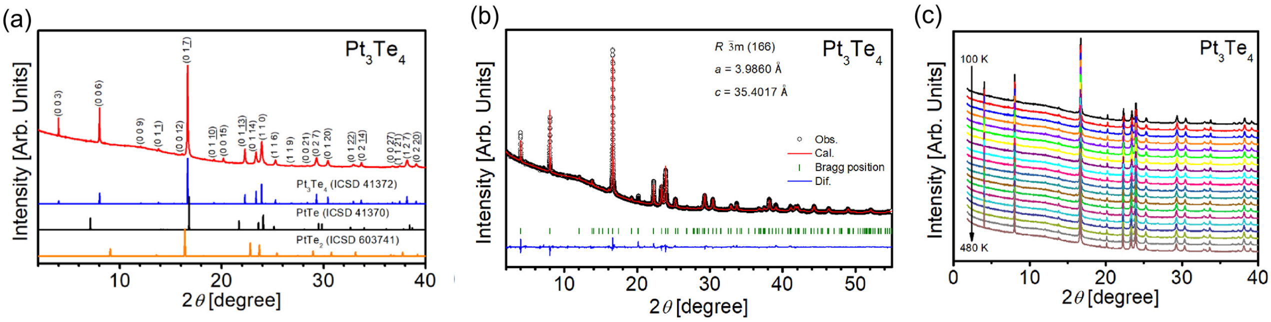

Figure S4. (a) The SXRD of the Pt_3Te_4 crystal at T=300 K. The simulated SXRD patterns of Pt_3Te_4, PtTe, and $PtTe_2$ are shown below for the sake of comparison. (b) The Rietveld refinement of the SXRD of Pt_3Te_4 crystal at T=300 K. (c) Selected SXRD patterns of Pt_3Te_4 in the temperature range of 100 – 480 K with an interval of 20 K.

S7. Transport measurements

For the Hall measurements, the Pt_3Te_4 crystal was mechanically exfoliated onto a Si/SiO_2 substrate with a 300nm oxide layer. The sample was then patterned by electron-beam lithography and metallized by electron beam deposition with Ti/Au 5/100 nm at background pressure of ~2 X 10^7 Torr. See the inset of Fig. R3 (a) for the topographic map of the device.
Hall measurements were taken at a temperature of 1.6 K and at magnetic field of 0-7 T. The measured temperature dependence of the longitudinal resistivity, and the B dependence of both the longitudinal and the Hall resistivity is shown in Fig. R3. We find the temperature coefficient of resistance to be almost constant ~0.5%/K [see the red line in panel a)].
The calculated electronic band structure of Pt_3Te_4 clearly shows the presence of both electron and hole pockets at the Fermi energy. Thus, the obtained Hall resistivity data is fitted by a two carrier (electrons and holes) model for the Hall conductivity, given by

$$\rho_{xy} = \frac{1}{e}\frac{(n_h\mu_h^2 - n_e\mu_e^2) + \mu_h^2\mu_e^2B^2(n_h - n_e)}{(n_h\mu_h + n_e\mu_e)^2 + \mu_h^2\mu_e^2B^2(n_h - n_e)^2}B, \qquad (1)$$

$$\rho_{xx} = \frac{1}{e}\frac{(n_h\mu_h + n_e\mu_e) + (n_e\mu_e\mu_h^2 + n_h\mu_h\mu_e^2)B^2}{(n_h\mu_h + n_e\mu_e)^2 + \mu_h^2\mu_e^2B^2(n_h - n_e)^2}, \qquad (2)$$

Remarkably, we find that in spite of the presence of both the carriers, the Hall resistivity varies linearly with the magnetic field and it has a negative slope. The negative slope confirms the dominating charge carriers to be the electrons. We estimate the density of electrons to be ~$2\cdot10^{21}$ cm^{-3}. The mobility of the electrons is found to be $\mu = 196.7\ \frac{cm^2}{V\,s}$.

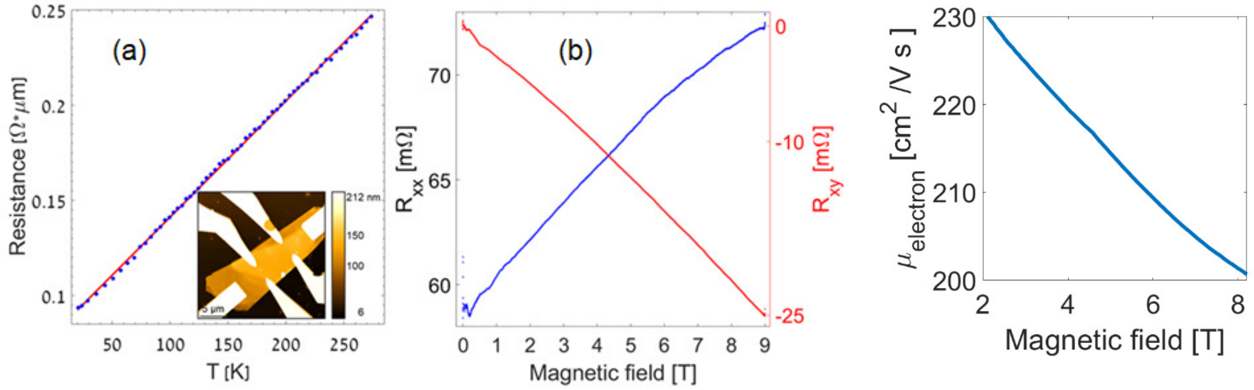

Figure S5. Temperature dependence Hall mobility and carrier concentration for Pt_3Te_4 measured at H = 6 T.

S8. Transmission electron microscopy (TEM)

TEM and HR-TEM images were obtained with FEI Tecnai G2 F30 at accelerating voltage of 300 kV. The bulk Pt_3Te_4 was grounded into powder and dissolved in ethanol for sample preparation.

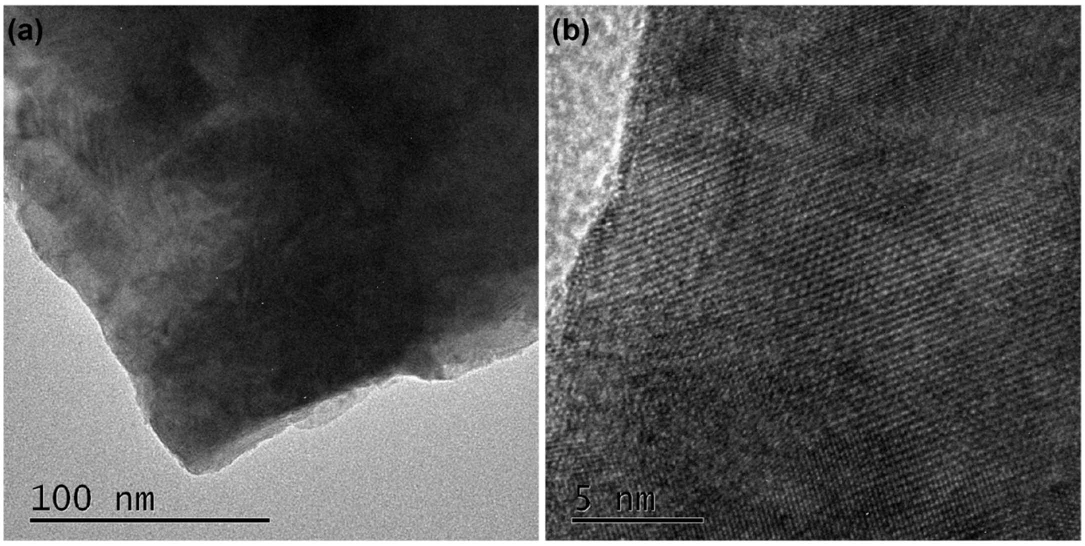

Figure S6. (a) TEM and (b) HR-TEM images of Pt_3Te_4.

S9. Scanning electron microscopy (SEM)

SEM experiments were performed with FEI Quanta 250 FEG at accelerating voltage of 10 kV. From the SEM images, the layered structure of pristine Pt_3Te_4 can be easily identified. After HER test, there is no obvious change can be found for Pt_3Te_4, and it remains the layered structure very well.

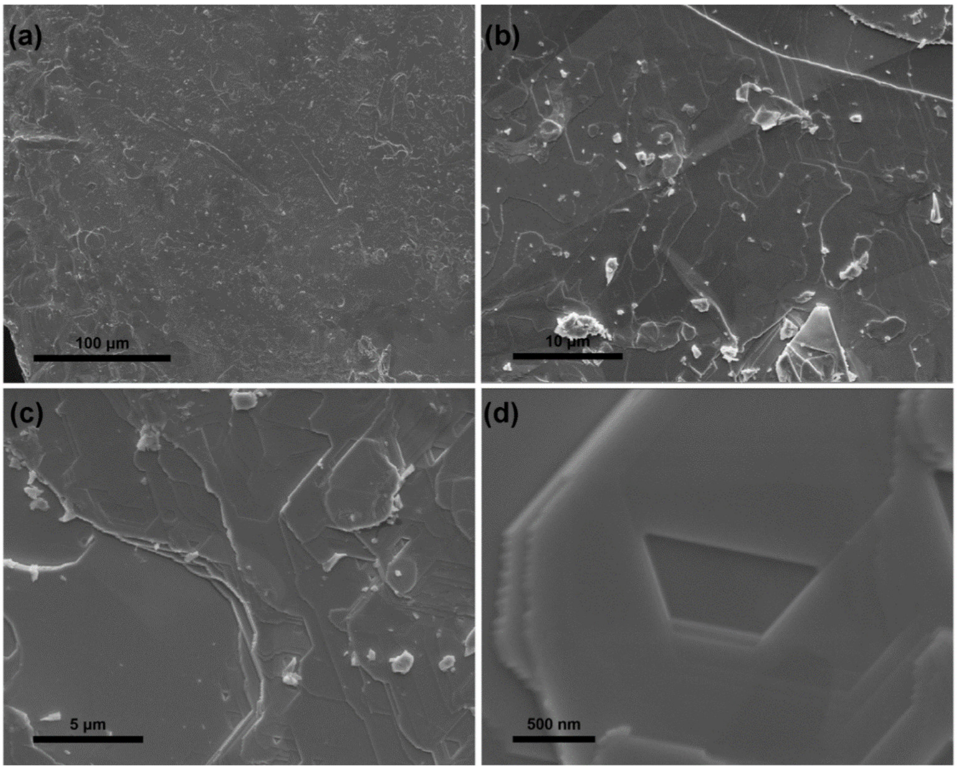

Figure S7. The typical SEM images of pristine Pt_3Te_4.

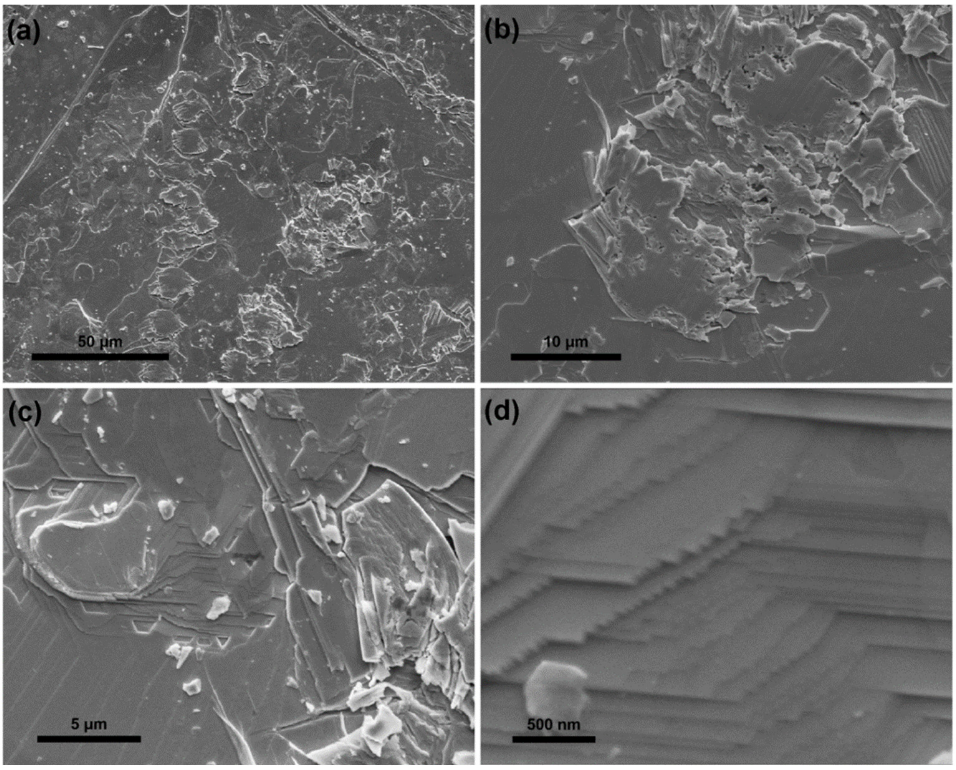

Figure S8. SEM images related to the morphological post-mortem analysis of the Pt_3Te_4-based electrode.

S10. CO poisoning: an assessment by HREELS

The adsorption of CO can be nicely followed by the HREELS technique, that is especially sensitive to CO adsorption (see our review for CO adsorption on catalytic surfaces [6] for more details), due to the high oscillating dipole. We carried out the experiment in specular conditions in order to maximize the sensitivity to dipole oscillations. These experiments were carried out with an Delta0.5 HREELS spectrometer by Specs GmbH, with a primary electron beam energy of 4 eV.

Explicitly, we dosed CO onto (i) Pt_3Te_4(001); (ii) $PtTe_2$(001); (iii) Ni(111); (iv) Pt_3Ni(111); and (v) Pt(111) surfaces. We obtained that, while Ni(111), Pt_3Ni(111) and Pt(111) are poisoned by CO, as evidenced by the observation of C-O stretching and the CO-substrate vibrations, $PtTe_2$ and Pt_3Te_4 are totally inert toward CO, even after prolonged CO exposure up to 10^{10} L.

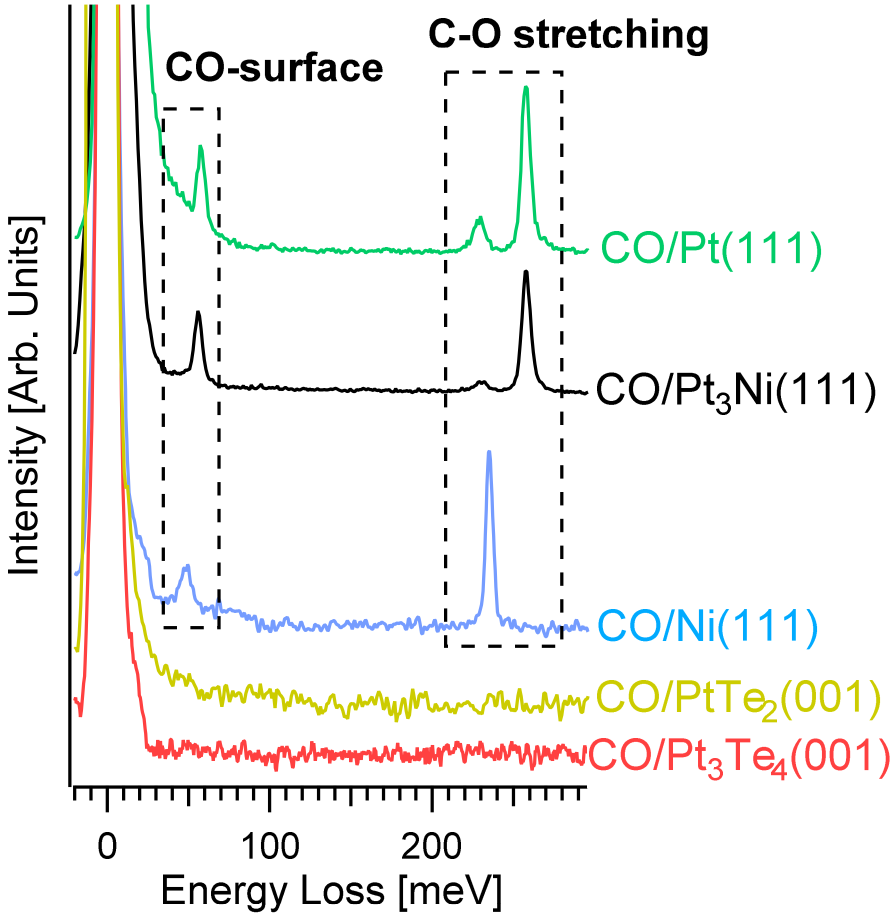

Figure S9. Vibrational spectra after having saturated with CO the surfaces of Pt_3Te_4(001), $PtTe_2$(001), Ni(111), Pt_3Ni(111) and Pt(111). The saturation has been reached at only 5, 10, and 8 L for Ni(111), Pt_3Ni(111) and Pt(111). On the other hand, no CO-derived features are achieved even after exposure to 10^{10} L on Pt_3Te_4(001) and $PtTe_2$(001). Specifically, the CO-derived features are the vibration of the whole CO molecule against the substrate at 50 meV [6-7] and the intramolecular C-O stretching [6], whose energy depends on the adsorption site: 230 meV for three-fold site, selectively occupied on Ni, while it is minoritary on Pt_3Ni(111) and Pt(111), and 250 meV for the on-top adsorption site, majoritary on both Pt_3Ni(111) and Pt(111).

S11. Determination of the thickness of the oxide skin

Assuming an ideal situation in which a uniform layer of TeO_2 is formed, we estimated the surface oxide thickness, d (Å) from Te-3d spectrum. The intensity ratio between oxidized species I_o and pristine surface I_m of Pt_3Te_4, using the following equation [8-9]:

$$d\ (\text{Å}) = \lambda_o \sin\theta \ln\left(\frac{N_m \lambda_m I_o}{N_o \lambda_o I_m} + 1\right)$$

where λ_o and λ_m are the effective attenuation lengths (EALs) of TeO_2 and Pt_3Te_4, respectively.

The peak intensities, I_o and I_m, where obtained from XPS data analysis of Te-3d core level, while the EAL values at the experimental electron energy of Te-3d were calculated with QUASES-IMFP-TPP2M Ver. 3.0, which is based on the Tanuma, Powel, Penn TPP-2M formula [10]. N_m and N_o are the volume densities of Te atoms in Pt_3Te_4 and TeO_2, respectively. θ is the angle between the sample surface and the axis of the electron analyzer, as depicted in Fig. S10.

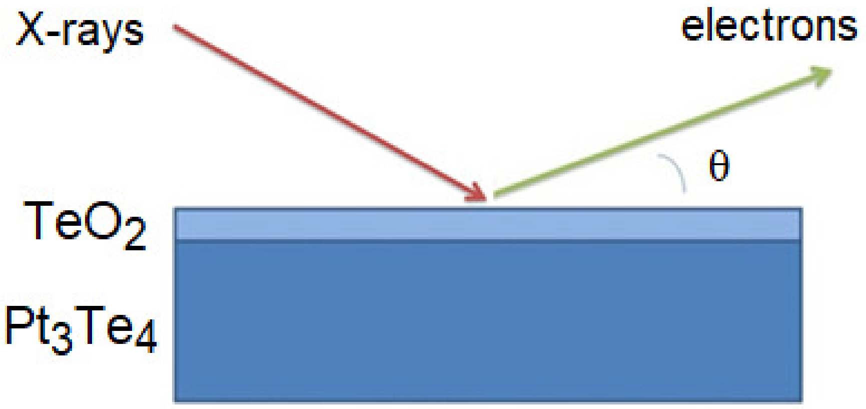

Figure S10. Geometry used for XPS measurements.

S12. Characterization of the sample after electrochemical treatment

To correlate the observed modifications in HER activity upon electrocatalytic treatment with changes in physicochemical and electronic properties, mitrofanovite modified by electrochemical treatments was characterized by means of XPS investigations (Fig. S11). Specifically, the oxidation treatment implies

the emergence of Te(0) (Te-3$d_{5/2}$ peak at 573.3 eV) and TeO_2 components (38% of the total spectral area with Te-3$d_{5/2}$ peak at 575.5 eV). In the reduced sample, the TeO_2 component is reduced by 70%. The analysis of the Pt-4f reveals that the oxidation treatment also introduces PtO_2 species (14% of the total spectra area, Pt-4$f_{7/2}$ at BE=74.0 eV). Corresponding microscopical data are reported in Fig. S12. It is important to point out that XPS data clarify that no Te dissolution is induced by electrochemical treatments, as evidenced by the quantitative analysis of the Pt/Te ratio.

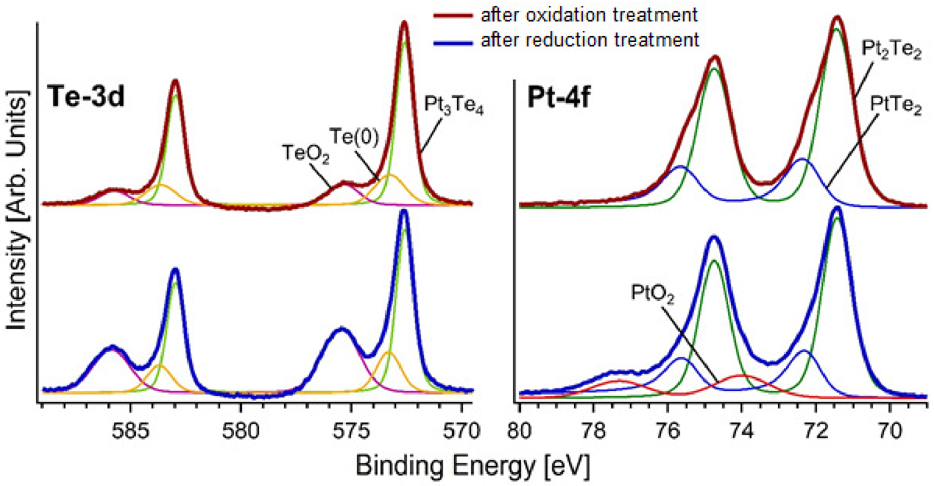

Figure S11. Core-level spectra of Te-3d and Pt-4f for Pt_3Te_4 after oxidized treatment (blue curve) and after reduced treatment (brown curve). The photon energy is 1486 eV (Al K_α) and the spectra are normalized to the maximum.

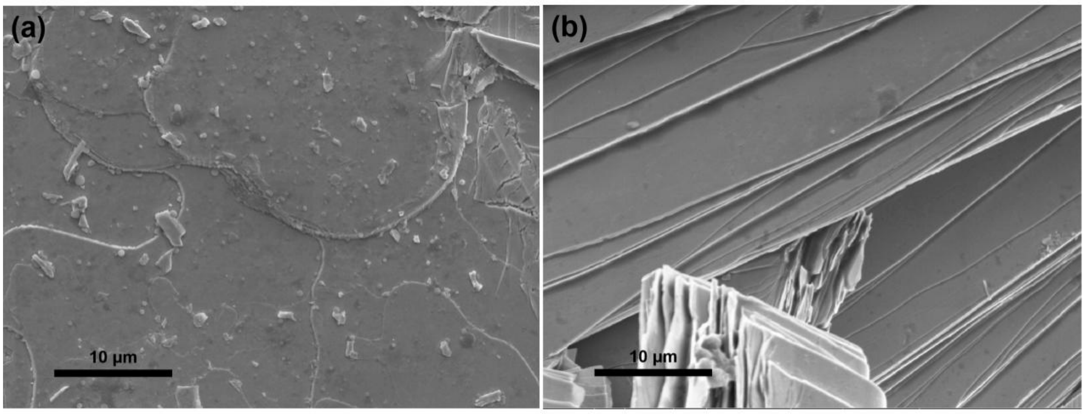

Figure S12. The typical SEM images of Pt_3Te_4 after (a) oxidation treatment and (b) reduction treatment.

S13. Band structure

To understand the topology of this system, we computed all four Z_2 topological invariants. As Pt_3Te_4 preserves inversion symmetry, we used the parity-based method developed by Fu-Kane [11] to calculate the topological invariant. By calculating the parity eigenvalues of all the filled bands, we find the Z_2 index to be (0;111). Since all the weak Z_2 indices are 1, while the strong index is 0, Pt_3Te_4 can be described as a weak topological metal.

Figure S13 reports the effects of spin-orbit coupling (SOC) on the band structure. The Brillouin zone is reported in Figure S14.

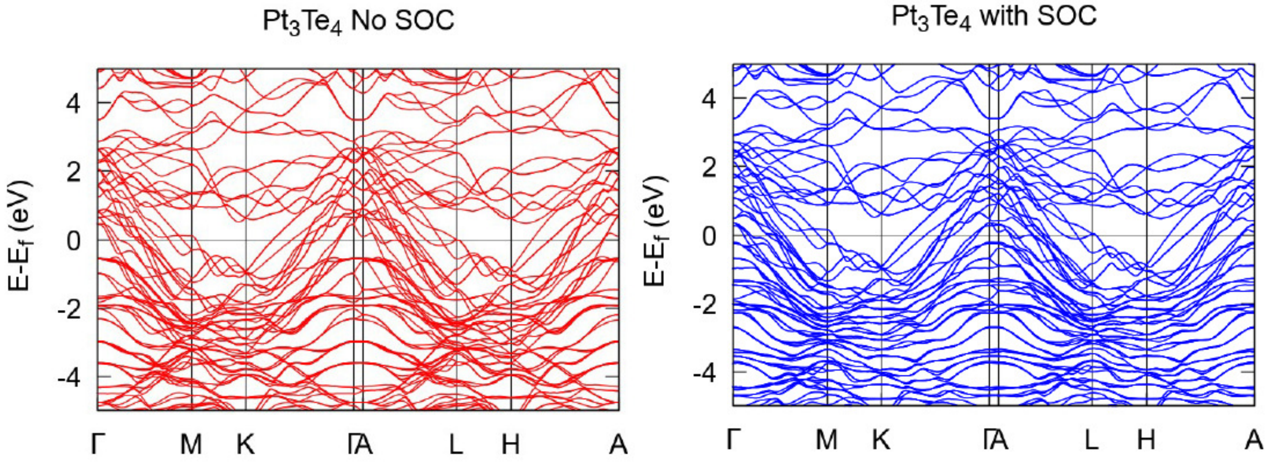

Figure S13. Band structure of mitrofanovite

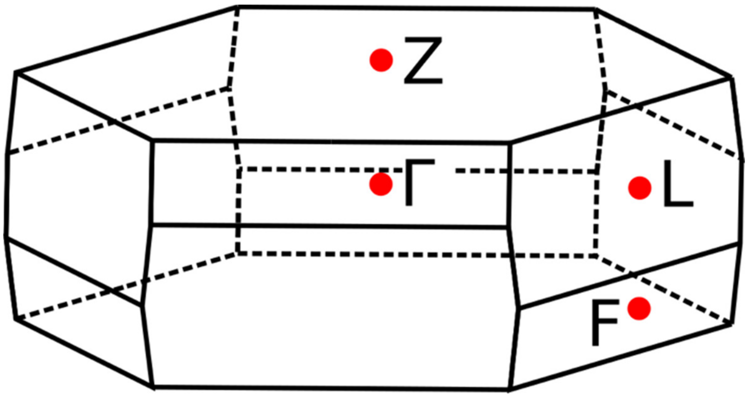

Figure S14. Brillouin zone

S14. Influence of Te vacancies on catalytic active: insights from DOS

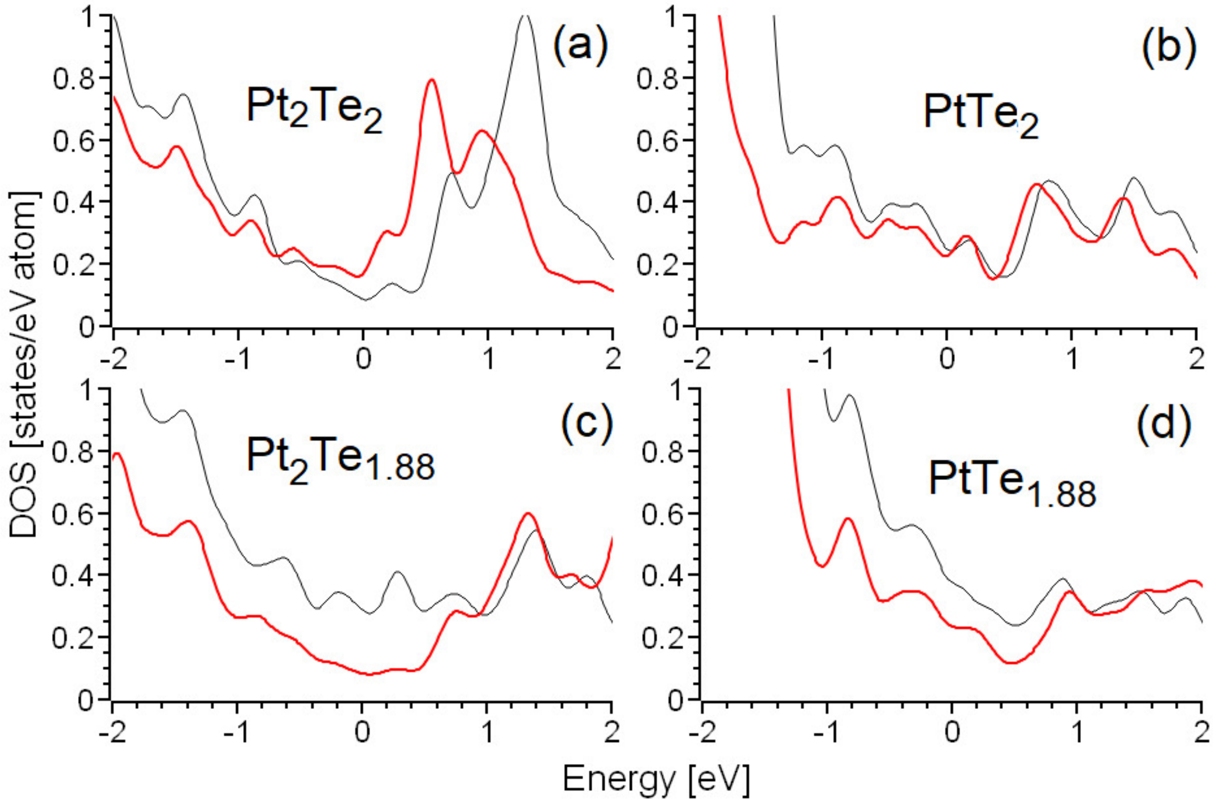

Figure S15. Partial densities of states for 5d shells of Pt atoms from surface layers of different types of considered surfaces before (black lines) and after (red lines) chemical adsorption of hydrogen atom. Fermi level set as zero.

S15. Optimized atomic structures of the adopted supercell

In Fig. S16 the optimized atomic structures of the adopted supercell is shown.

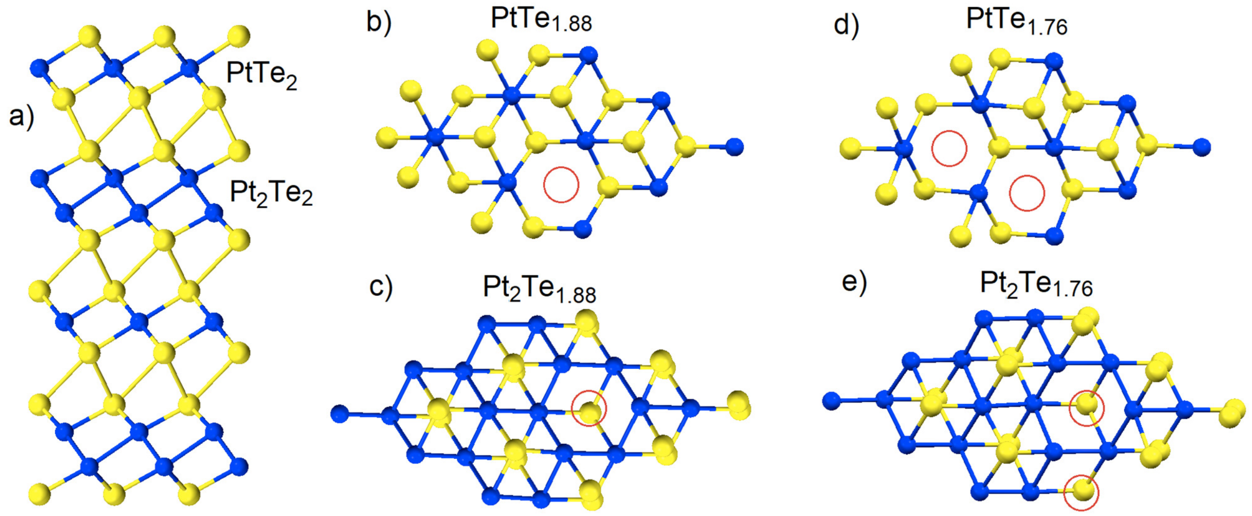

Figure S16. (a) Optimized atomic structure of Pt_3Te_4 slab used for calculations and top views of surface layers with (b,c) single and (d,e) double Te-vacancies in (b,d) $PtTe_2$ and (d,e) Pt_2Te_2 surfaces. Note that on panels (b-e) atoms are shown only on the surface layers, while atoms of sublayers are not shown for clarity of picture.

S16. Comparison with previous papers on mitrofanovite

In the in Ref. [12], authors reported results of the calculations for $PtTe_2$ and Pt_2Te_2 monolayers, which constitute the sub-units of Pt_3Te_4. Note that in these calculations, the contributions from interlayer interactions are not considered at all. It is evident that mitrofonovite Pt_3Te_4 is not the simple addition of $PtTe_2$ and Pt_2Te_2 units not interacting between them.

Therefore, results reported in discussed paper are actually inappropriate for understanding catalytic properties of mitrofanovite, which are obviously ruled by the surface properties of bulk crystals.

In addition, in the paper in Ref. [12] calculations for the monolayer $PtTe_2$ and Pt_2Te_2 have been performed without optimization of lattice parameters, which instead is essential for the description of chemical properties of flexible membranes. Thus, values reported in Ref. [12] are irrelevant also with free-standing monolayers.

Therefore, we can conclude that unfortunately results in Ref. [12] are not reliable.

Note that the above-mentioned technical aspects regarding the modelling of layered systems are known about one decade. Since pioneering experimental reports about oxidation of mono-, bi- and multilayers of graphene at different temperatures [13] and special pathways of functionalization of graphene on SiC [14], taking into account of the presence of sublayers and flexibility of membranes is essential for building of proper model (see for review Ref. [15]). Turning from graphene to other layered systems also demonstrate importance of adopting proper models for DFT-based calculations. The most spectacular examples of this issue are InSe and GaSe, for which monolayers are chemically stable [16-17] in contrast to bulk materials (see our recent work [18]).

Concerning HER modelling, obtained results in our case (1.06 eV/H^+ for $PtTe_2$-termination and 0.65 eV/H_+ for Pt_2Te_2-termination) are smaller than those calculated for monolayers (1.20 and 0.89 eV/H^+, respectively) [12]. The difference in the values calculated for slab and monolayers demonstrates the significant influence of interlayer non-covalent interactions on chemical properties. Therefore, adopting a theoretical model based on monolayers as in Ref. [12] is improper for the description of catalytic properties of the mitrofanovite surface. Note that even the calculations in Ref. [12], performed for monolayers without optimization of lattice parameters, also indicate decreasing of the free energy of this step to +0.17 eV/H^+ for $PtTe_2$ and +0.29 eV/H^+ for Pt_2Te_2 [12]. In the presence of the vacancies, the effect of sublayers on energetics of adsorption is more dramatic than in the case of monolayer (see discussion above), corresponding to the appearance of additional structural degrees of freedom of the top layer after defects formation.

Concerning band structure in Ref. [12], we note that the authors consider the band structure and formation energy of *monolayers* of $PtTe_2$ and Pt_2Te_2 [see Fig. 5 (a)-(c) in Ref. [12]) to describe the electronic and stability properties of bulk Pt_3Te_4. Even if we consider super-simplified model of the outermost (at the surface) unit cell of Pt_3Te_4 – it has multiple layers of $PtTe_2$ and Pt_2Te_2 which have significant interlayer interactions between them. Extrapolating the electronic properties of monolayers for describing the bulk properties can lead to wrong conclusions such as Pt_3Te_4 being an insulator (see Fig. 5(a) in Ref. [2]), while actually it is a metal with a large carrier concentration. This also leads to wrong estimation of the formation energy.

Concerning experimental data, in Figure 4a of the paper in Ref. [12], in correspondence of an overpotential of 0 V vs RHE, both the current densities for Pt_3T_4 nanocrystal and Pt/C samples are clearly larger than 0, which is quite puzzling. This result indicates that the reference electrode they used may not be calibrated correctly, and thus the HER data they reported are not really solid.

S17. Nyquist plots

The Nyquist plots of the pristine and the electrochemically treated Pt_3Te_4 were tested in 0.1 M KCl solution containing 5mM $[Fe(CN)_6]^{3-/4-}$ (SI, Fig. S17). The charge transfer resistance (R_{ct}) of the pristine, oxidized and reduced Pt_3Te_4 samples are fitted to be 102.9, 84.2 and 76.5 Ω, respectively. This result indicates the slightly faster charge transfer kinetics on the oxidized and reduced Pt_3Te_4 than on the pristine Pt_3Te_4, which agrees with the cyclic voltammetry test.

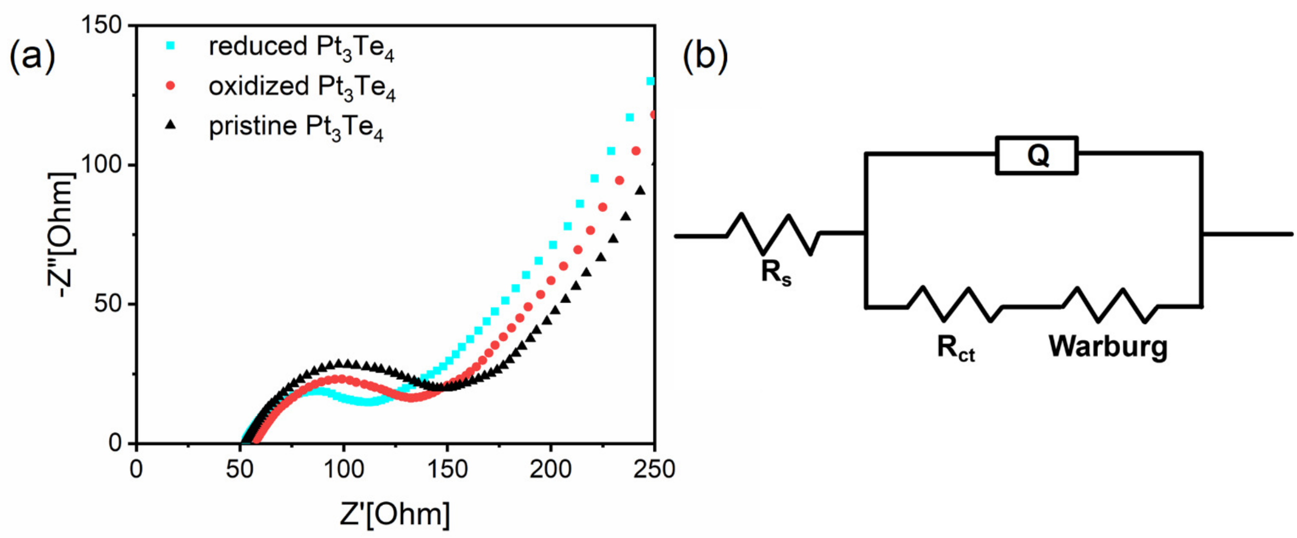

Figure S17. (a) Nyquist plots of the pristine and the electrochemically treated Pt_3Te_4 in 0.1 M KCl solution containing 5mM $[Fe(CN)_6]^{3-/4-}$ at open-circuit potential with an amplitude of 5 mV, and the frequency range is 10^6-0.01 Hz. (b) The equivalent circuit for the Nyquist plots fitting, in which R_s is the uncompensated solution resistance, Q is the constant phase element component, R_{ct} represents the charge transfer resistance, and Warburg refers to the Warburg resistance.

References:

1. Kresse, G.; Joubert, D., From Ultrasoft Pseudopotentials to the Projector Augmented-Wave Method. *Phys. Rev. B* **1999**, *59*, 1758, doi: 10.1103/PhysRevB.59.1758
2. Blöchl, P. E.; Jepsen, O.; Andersen, O. K., Improved Tetrahedron Method for Brillouin-Zone Integrations. *Phys. Rev. B* **1994**, *49*, 16223-16233, doi: 10.1103/physrevb.49.16223
3. Kresse, G.; Furthmüller, J., Efficient Iterative Schemes for ab initio Total-Energy Calculations Using a Plane-Wave Basis Set. *Phys. Rev. B* **1996**, *54*, 11169-11186, doi: 10.1103/PhysRevB.54.11169
4. Perdew, J. P.; Burke, K.; Ernzerhof, M., Generalized Gradient Approximation Made Simple. *Phys. Rev. Lett.* **1996**, *77*, 3865-3868, doi: 10.1103/PhysRevLett.77.3865
5. Togo, A.; Tanaka, I., First Principles Phonon Calculations in Materials Science. *Scr. Mater.* **2015**, *108*, 1-5, doi: 10.1016/j.scriptamat.2015.07.021
6. Politano, A.; Chiarello, G., Vibrational Investigation of Catalyst Surfaces: Change of the Adsorption Site of CO Molecules Upon Coadsorption. *J. Phys. Chem. C* **2011**, *115*, 13541-13553, doi: 10.1021/jp202212a
7. Politano, A.; Formoso, V.; Agostino, R. G.; Colavita, E.; Chiarello, G., Influence of Co Adsorption on the Alkali-Substrate Bond Studied by High-Resolution Electron Energy Loss Spectroscopy. *Phys. Rev. B* **2007**, *76*, 233403, doi: 10.1103/PhysRevB.76.233403
8. Alexander, M. R.; Thompson, G. E.; Zhou, X.; Beamson, G.; Fairley, N., Quantification of Oxide Film Thickness at the Surface of Aluminium Using XPS. *Surf. Interface Anal.* **2002**, *34*, 485-489, doi: 10.1002/sia.1344

9. Sciortino, L.; Lo Cicero, U.; Magnano, E.; Píš, I.; Barbera, M., *Surface Investigation and Aluminum Oxide Estimation on Test Filters for the Athena X-IFU and WFI Detectors*; SPIE, 2016; Vol. 9905.
10. Tanuma, S.; Powell, C. J.; Penn, D. R., Calculations of Electron Inelastic Mean Free Paths. V. Data for 14 Organic Compounds over the 50–2000 eV Range. *Surf. Interface Anal.* **1994**, *21*, 165-176, doi: 10.1002/sia.740210302
11. Kane, C. L.; Mele, E. J., Z_2 Topological Order and the Quantum Spin Hall Effect. *Phys. Rev. Lett.* **2005**, *95*, 146802, doi: 10.1103/PhysRevLett.95.146802
12. Bae, D.; Park, K.; Kwon, H.; Won, D.; Ling, N.; Baik, H.; Yang, J.; Park, H. J.; Cho, J.; Yang, H., Mitrofanovite, Layered Platinum Telluride, for Active Hydrogen Evolution. *ACS Appl. Mater. Interfaces* **2021**, *13*, 2437–2446, doi: 10.1021/acsami.0c16098
13. Liu, L.; Ryu, S.; Tomasik, M. R.; Stolyarova, E.; Jung, N.; Hybertsen, M. S.; Steigerwald, M. L.; Brus, L. E.; Flynn, G. W., Graphene Oxidation: Thickness-Dependent Etching and Strong Chemical Doping. *Nano Lett.* **2008**, *8*, 1965-1970, doi: 10.1021/nl0808684
14. Hossain, M. Z.; Johns, J. E.; Bevan, K. H.; Karmel, H. J.; Liang, Y. T.; Yoshimoto, S.; Mukai, K.; Koitaya, T.; Yoshinobu, J.; Kawai, M.; Lear, A. M.; Kesmodel, L. L.; Tait, S. L.; Hersam, M. C., Chemically Homogeneous and Thermally Reversible Oxidation of Epitaxial Graphene. *Nat. Chem.* **2012**, *4*, 305-309, doi: 10.1038/nchem.1269
15. Boukhvalov, D., DFT Modeling of the Covalent Functionalization of Graphene: From Ideal to Realistic Models. *RSC Adv.* **2013**, *3*, 7150-7159, doi: 10.1039/C3RA23372C
16. Politano, A.; Chiarello, G.; Samnakay, R.; Liu, G.; Gurbulak, B.; Duman, S.; Balandin, A. A.; Boukhvalov, D. W., The Influence of Chemical Reactivity of Surface Defects on Ambient-Stable InSe-Based Nanodevices. *Nanoscale* **2016**, *8*, 8474-8479, doi: 10.1039/C6NR01262K
17. Petroni, E.; Lago, E.; Bellani, S.; Boukhvalov, D. W.; Politano, A.; Gürbulak, B.; Duman, S.; Prato, M.; Gentiluomo, S.; Oropesa-Nuñez, R.; Panda, J. K.; Toth, P. S.; Del Rio Castillo, A. E.; Pellegrini, V.; Bonaccorso, F., Liquid-Phase Exfoliated Indium–Selenide Flakes and Their Application in Hydrogen Evolution Reaction. *Small* **2018**, *14*, 1800749, doi: 10.1002/smll.201800749
18. D'Olimpio, G.; Nappini, S.; Vorokhta, M.; Lozzi, L.; Genuzio, F.; Menteş, T. O.; Paolucci, V.; Gürbulak, B.; Duman, S.; Ottaviano, L.; Locatelli, A.; Bondino, F.; Boukhvalov, D. W.; Politano, A., Enhanced Electrocatalytic Activity in GaSe and InSe Nanosheets: The Role of Surface Oxides. *Adv. Funct. Mater.* **2020**, *30*, 2005466, doi: 10.1002/adfm.202005466
